# Novel 2-Hydroselenonicotinonitriles and Selenopheno[2, 3-b]pyridines: Efficient Synthesis, Molecular Docking-DFT Modeling, and Antimicrobial Assessment

**DOI:** 10.3389/fchem.2021.672503

**Published:** 2021-05-10

**Authors:** Magda H. Abdellattif, Adel A. H. Abdel-Rahman, Mohamed Mohamed Helmy Arief, Samar M. Mouneir, Amena Ali, Mostafa A. Hussien, Rawda M. Okasha, Tarek H. Afifi, Mohamed Hagar

**Affiliations:** ^1^Department of Chemistry, College of Science, Deanship of Scientific Research, Taif University, Taif, Saudi Arabia; ^2^Chemistry Department, Faculty of Science, Menoufia University, Shibin El Kom, Egypt; ^3^Chemistry Department, Faculty of Science, Benha University, Benha, Egypt; ^4^Department of Pharmacology, Faculty of Veterinary Medicine, Cairo University, Giza, Egypt; ^5^Department of Pharmaceutical Chemistry, College of Pharmacy, Taif University, Taif, Saudi Arabia; ^6^Department of Chemistry, Faculty of Science, King Abdulaziz University, Jeddah, Saudi Arabia; ^7^Department of Chemistry, Faculty of Science, Port Said University, Port Said, Egypt; ^8^Department of Chemistry, Faculty of Science, Taibah University, Medina, Saudi Arabia; ^9^Chemistry Department, College of Sciences, Taibah University, Yanbu, Saudi Arabia; ^10^Chemistry Department, Faculty of Science, Alexandria University, Alexandria, Egypt

**Keywords:** selenopyridines, antimicobaterial, molecular docking, DFT calculations, one pot synthesis

## Abstract

Selenium containing heterocyclic compounds gained great interest as bioactive molecules as of late. This report explores the design, synthesis, characterization, and antimicrobial screening of new pyridine derivatives endowed with selenium moieties. A one-pot multicomponent system with a solvent-free, microwave irradiation environment was employed to afford this series. The spectroscopic techniques were exploited to verify the structures of the synthesized derivatives. Additionally, the agar diffusion method was employed to determine the antimicrobial activity of all the desired compounds. Of all the synthesized molecules, **9b**, **12b**, **14f**, and **16d** exhibited well to remarkable antibacterial and antifungal activities. Moreover, derivative **14f** demonstrated the most potent antibacterial and antifungal performance. The results were also supported by molecular docking studies, utilizing the MOE (molecular operating environment) which revealed the best binding mode with the highest energy interaction within the binding pocket. Lastly, theoretical DFT calculations were carried out in a gas phase at B3LYP 6-311G (d,p) basis set to predict the molecular geometries and chemical reactivity descriptors. DFT results have been used to illustrate that molecular docking findings and biological activity assessments.

## Introduction

Microbial infection is one of the serious threats to human lives and causes major global public health issues. In the current scenario, there is a steady rise in the incidences of infectious diseases due to the rapid resistance of microbial strains to existing antimicrobial agents (Abdellattif, 2016; Al-Blewi et al., 2018; Fonkui et al., 2019). Thus, exploration of more selective, potent and less toxic antimicrobial agents has become a challenging task for researchers. Therefore, pyridone and its derivatives have attracted a great deal of interest due to their promising pharmacological activities such as antibacterial, antifungal (Fassihi et al., 2009); anti-HIV (Parreira et al., 2001); antitumor (Hasvold et al., 2003); anti-hepatitis B (Lv et al., 2010); anaplastic lymphoma kinase inhibitors (Li et al., 2006); antituberculotic agents (Ng et al., 2015); and anti Pim-1 kinase activities (Fujita et al., 2005; Cheney et al., 2007).

2-Pyridones are multifaceted compounds that are present in numerous bioactive natural products and pharmaceutical compounds (Gorobets et al., 2004; Mijin et al., 2014). Despite the availability of several conventional methods to synthesize pyridone derivatives or its use, solvent-free and environmentally safe green approaches are highly demanded as influential procedures from both the economical and synthetic point of view (Burger et al., 2002; Wathey et al., 2002; Kappe et al., 2008; Grundas, 2011; Mijin et al., 2014; Helmy et al., 2015; Poudel et al., 2015; Abdellattif et al., 2017; Hagar et al., 2020b). Pyridone derivatives also emerged as valuable building blocks for the synthesis of bioactive natural products as well as a versatile synthon for the synthesis of diverse nitrogen-containing heterocyclic compounds like β-lactams, indolizidine alkaloid, quinolizidines, and piperidines (Burger et al., 2002; Potewar et al., 2008).

In the recent years, selenium compounds have gained substantial interest not only for their importance in synthetic chemistry but also for their biological properties (Burling and Goldstein, 1992; Lobinski et al., 2000; Koketsu et al., 2002; Abdel-Hafez, 2008; Potewar et al., 2008; Abdel-Lattif et al., 2014; Al-Rubaie et al., 2014). Furthermore, the incorporation of selenium functionalities into organic scaffolds reported to permit alteration of their chemical and biological activities (Al-Smadi and Al-Momani, 2008). Many studies described the antimicrobial activity of selenium containing compounds specifically against several strains of bacteria causing nosocomial infections or forming biofilm, such as *Escherichia coli* and *Staphylococcus aureus* (Mosolygó et al., 2019). Moreover, selenium containing heterocyclic compounds are well-known for their antibacterial, antifungal, antiviral, antioxidant, anti-inflammatory, antileishmanial, and antimutagenic properties (Leyck and Parnham, 1990; Tapiero et al., 2003; Al-Smadi and Al-Momani, 2008; Maslat et al., 2010; Díaz et al., 2019; Spengler et al., 2019).

Based on the aforementioned facts and as an extension of our studies, a new synthetic approach has been developed for the synthesis of pyridine derivatives by incorporating selenium in its nucleus, employing a green microwave irradiation method. In the present study, 2-hydroselenonicotinonitrile and selenopheno[2, 3-b] pyridine derivatives were designed, synthesized, and explored for their antibacterial and antifungal activities. On the other hand, the advancement of computer-based technologies has progressively allowed simulating the dynamic nature of the binding event. Molecular docking is an attractive component of the drug discovery to recognize drug biomolecular interactions for the rational drug design. Therefore, molecular docking simulation was also performed for the newly synthesized compounds to find the potential binding mode and interaction energy.

**Figure 1:**
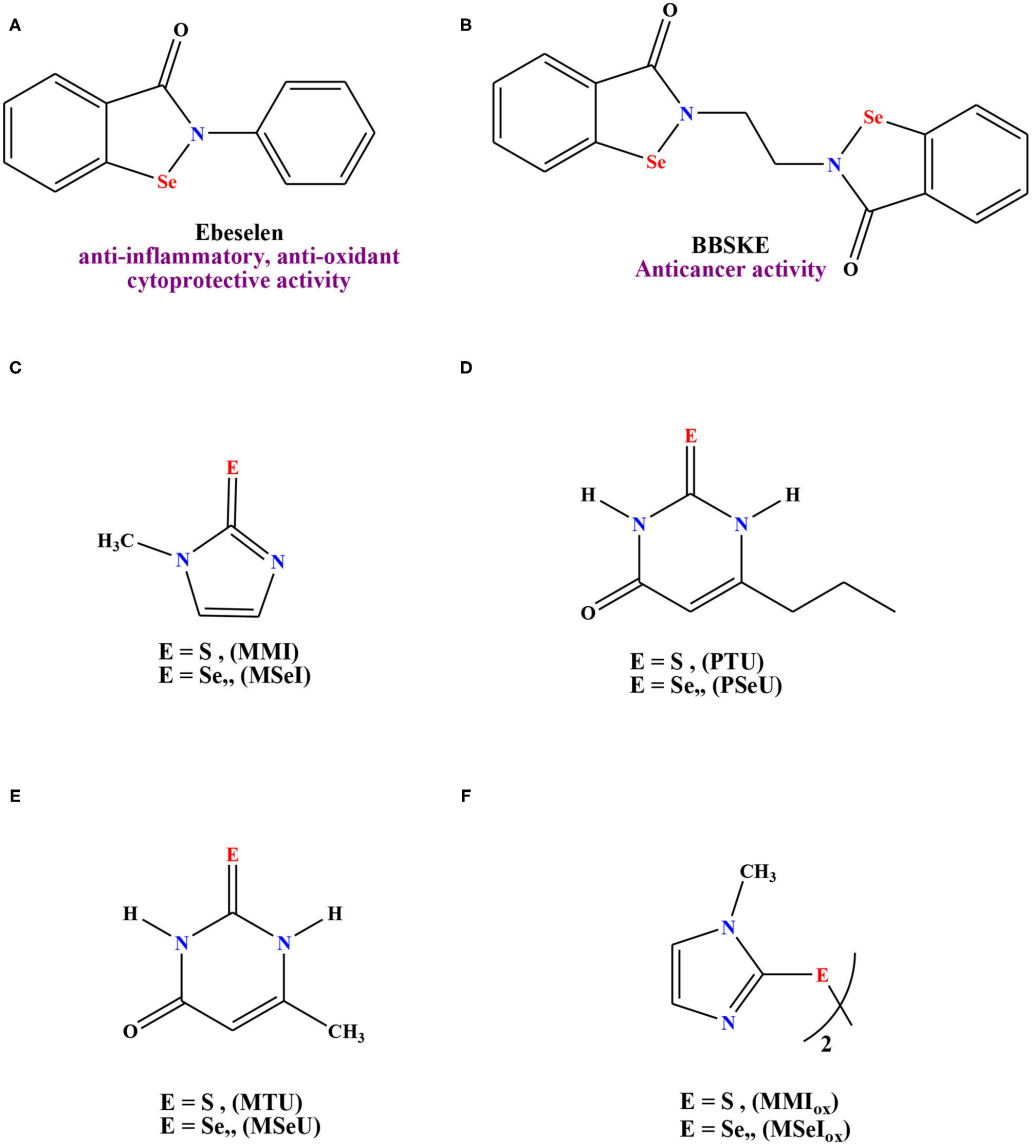
Reference drugs containing Selenium moieties and their sulfur analouges. **(A)** Ebeselen, **(B)** 1,2-[bis(1,2-benzisoselenazolone-3(2H)-ketone)]ethane (BBKSE), **(C)** methimazole derivatives, **(D)** 6-n-propyl-2-thio-/seleno-uracil, **(E)** 6-methyl-2-thio-/seleno-uracil, **(F)** dimer of methimazole derivatives.

Figure 1 illustrates the rational study of selected selenium containing compounds with demonstrated medicinal performance. Case in point, Abselen (**A**) is a renowned drug with antioxidant and anti-inflammatory features that is being employed in the treatment for bipolar disorder (Singh et al., 2013), hearing loss (Kil et al., 2007), and reperfusion injury (Yamaguchi et al., 1998; Parnham and Sies, 2000). Moreover, this molecule recently displayed a promising inhibition effect toward COVID-19 (Ewelina et al., 2021). Selenium derivative (**B**) has been established as an anticancer agent, particularly for the tongue and prostate adenocarcinoma (Xing et al., 2008). In addition, other selenium molecules have been employed as efficient antithyroid drugs in comparison with their sulfur analogs, compounds (**C-F**), Figure 1 (Roy and Mugesh, 2008).

## Results and Discussion

### Chemistry

**Figure 2:**
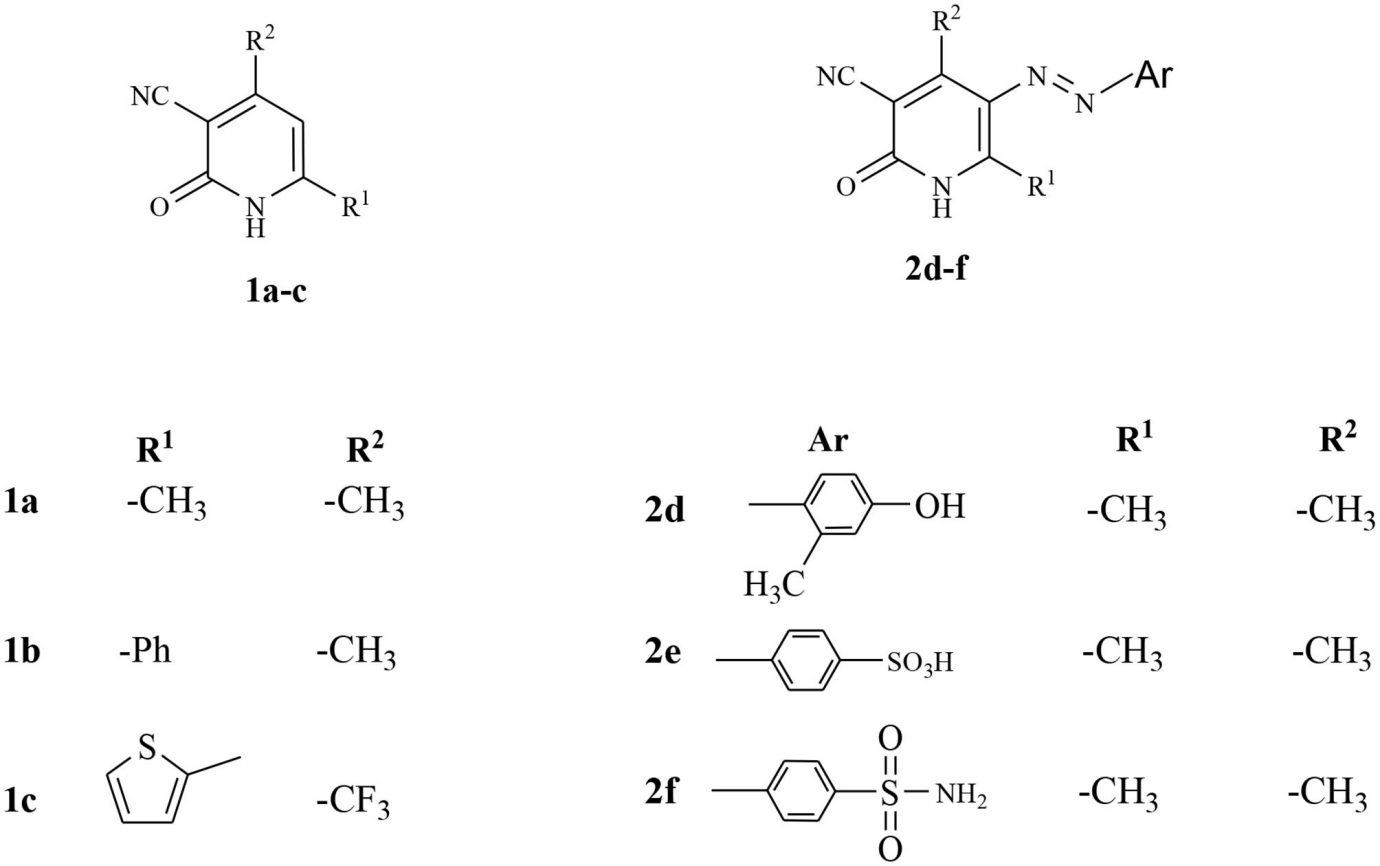
The utilized precursors derivatives (Abdellattif et al., 2017).

**Scheme 1:**
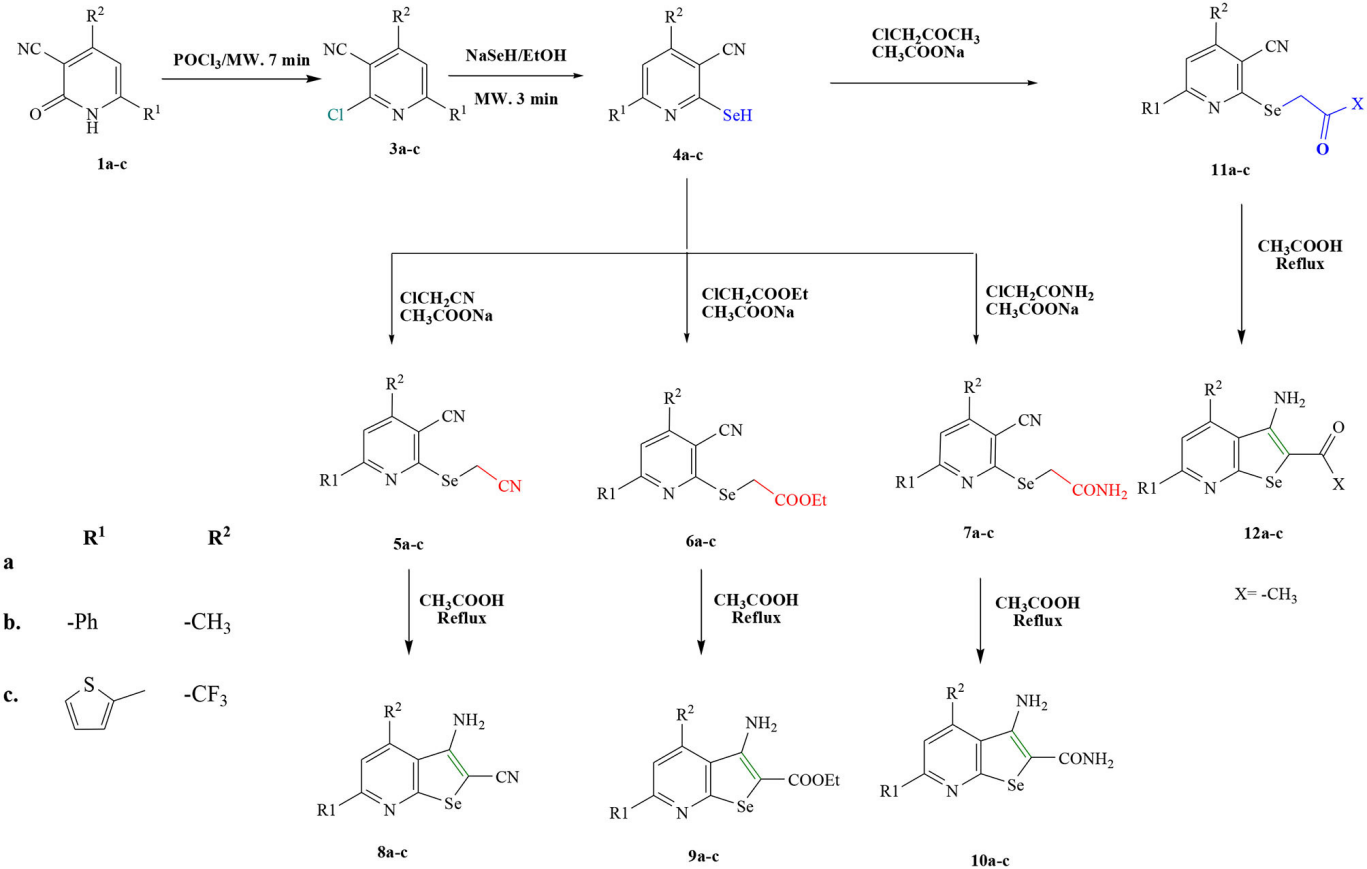
Synthesis of selenopheno[2, 3-b]pyridines **(4-12a-c)**.

**Scheme 2:**
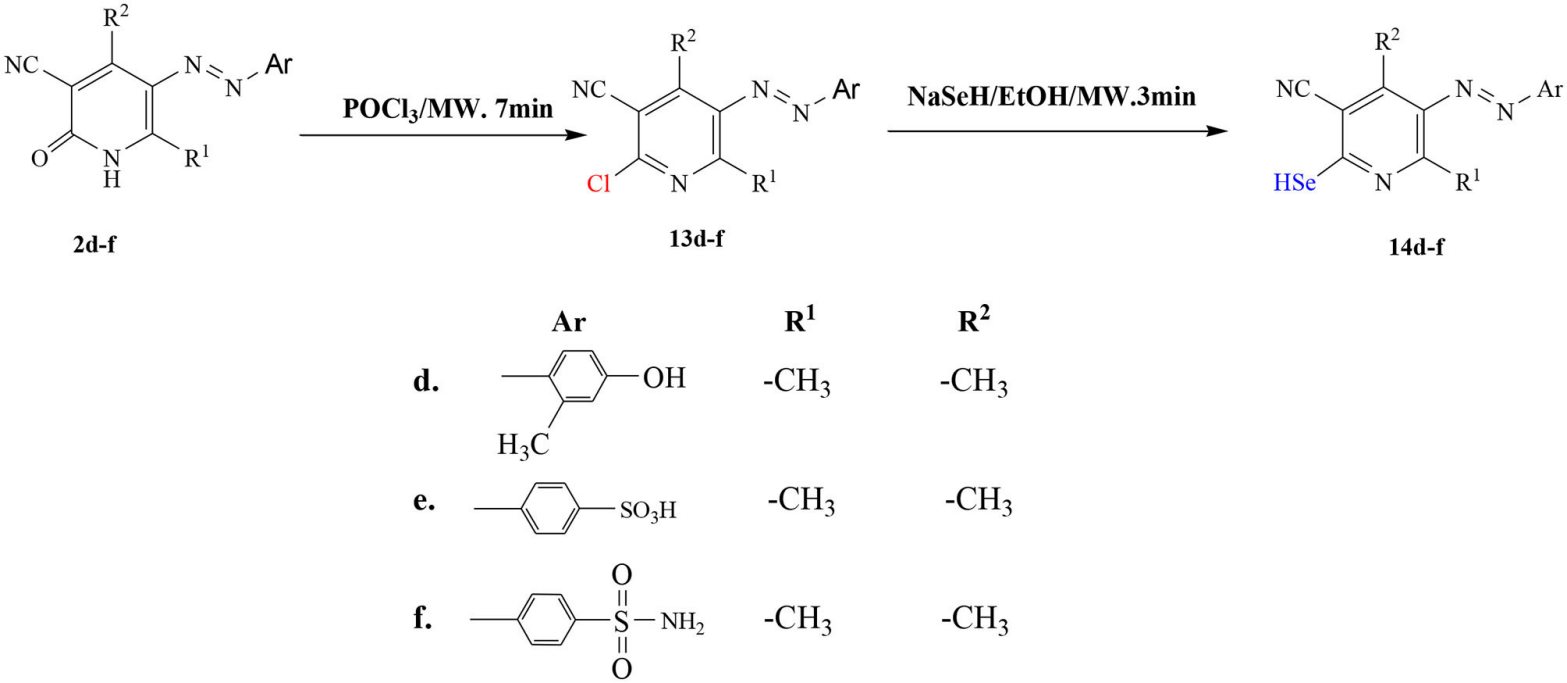
Synthesis of hydroselenonicotinonitrile **14d-f**.

**Scheme 3:**
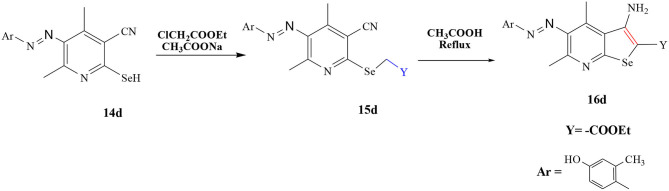
Synthesis of selenopheno[2, 3-b]pyridine derivative **16d**.

The starting materials, **1a-c** and **2d-f**, demonstrated in Figure 2, were synthesized as previously reported in literature (Abdellattif et al., 2017). Utilizing these precursors instigated the synthetic strategy to obtain the title compounds **8a-c, 9a-c, 10a-c**, **12a-c**, **14d-f**, and **16d** in excellent yield. The synthetic reaction sequence of the new molecules is delineated in Schemes 1–3. The structural identity of the synthesized compounds was confirmed utilizing the spectral data (IR, ^1^H NMR, ^13^C NMR, and mass) and the elemental analysis, which were in full harmony with the described structures. The desired compounds were obtained in excellent yields ranging from 89 to 94% after recrystallization with ethanol/dimethylformamide (DMF).

In general, the IR spectra displayed peaks at around 3,500–3,550 (OH, hydroxyl), 3,210–3,235 (N-H), 3,300, 3,400 (NH_2_), 2,970–3,048 (ArH), 2,850–2,910 (CH_2_), 1,650–1,720 (C=O), and 2,220–2,250 (C-N). All the respective protons of the ^1^H NMR spectra of the newly synthesized compounds were confirmed in accordance with their chemical shifts (δ) and multiplicities. The synthesized compounds exhibited singlets around δ 1.70–2.04 ppm, 4.32 and 5.1–5.4 ppm, which could be accounted for the methyl, hydroxyl, and amine protons, respectively. Meanwhile, the aromatic protons resonated in the range of δ 6.45–7.8, whereas the thienyl protons appeared in the 7.3–7.5 ppm. The ^13^C NMR spectra showed carbon signals corresponding to pyridone derivatives and aromatic rings. For instance, the ^13^C NMR spectra displayed peaks at δ 206–169 ppm for the carbonyl group, δ 125–135 ppm for the thienyl carbon, δ 89–143 ppm for the indenyl carbon, δ 217 ppm for CF_3_, 116–118 for C-N, and δ 83–166 ppm for the aromatic carbon. The peaks of the mass spectra (ESI-MS) appeared at definite *m/z* value according to the molecular formula of the compounds. The results of elemental analysis were within ±0.4% deviation as compared to the theoretical values for each element analyzed (C, H, N, and S).

An in-depth, detailed explanation of the newly synthesized molecules' characterization is presented in the experimental segment.

#### DFT Molecular Geometry

**Figure 3:**
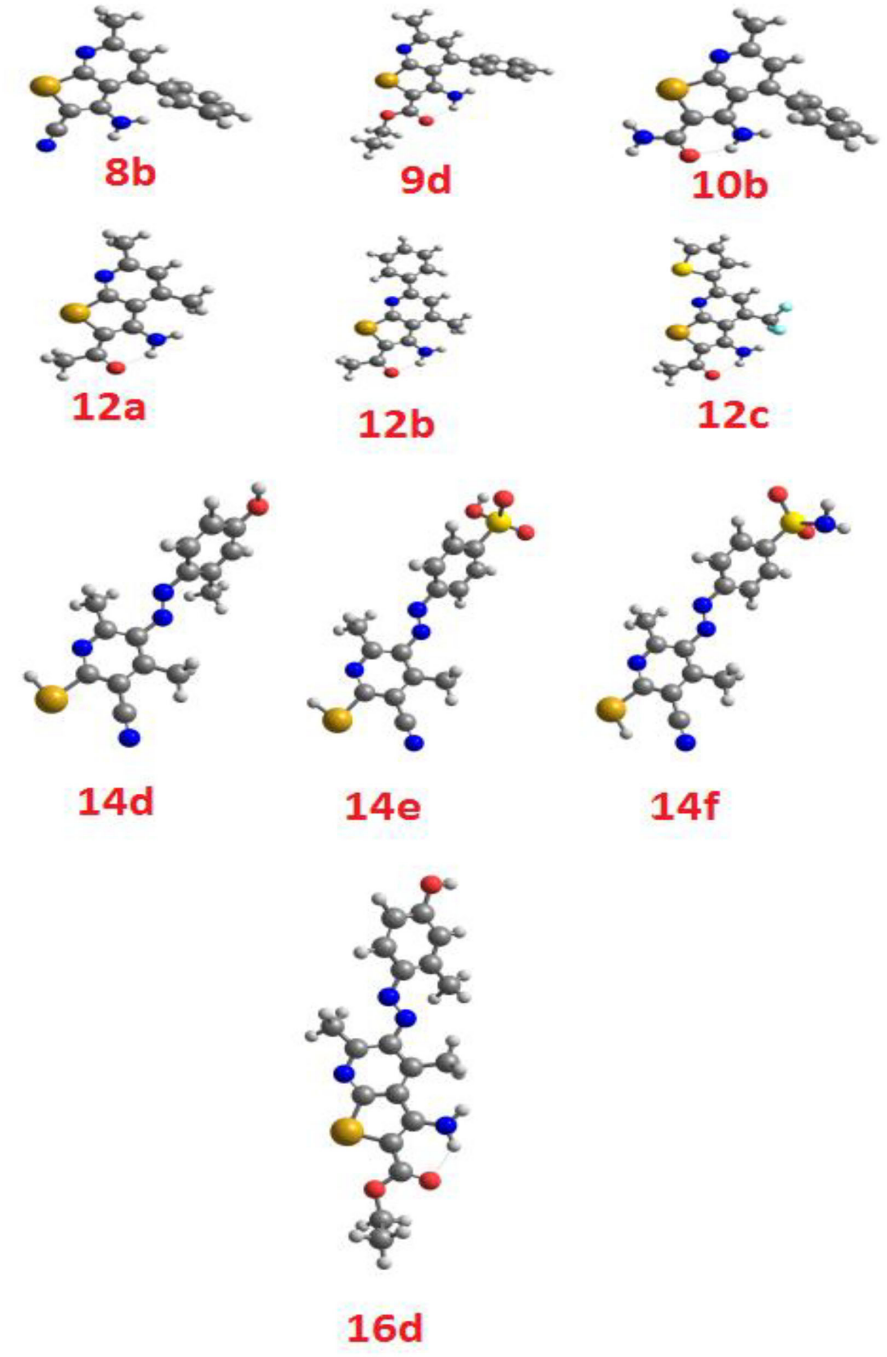
Optimized geometrical structures of the investigated compounds.

The theoretical DFT stimulation was achieved in a gas phase at B3LYP 6-311G (d,p) basis set implemented into the Gaussian 9. This included the prediction of the geometrical optimization on each prepared compound to determine the minimum energy molecular structure, followed by the frequency calculation at the optimum geometrical structural during which many thermochemical parameters were also calculated. All optimized geometrical structures of the investigated compounds are proved to be stable due to the absence of the imaginary frequency. The results of the DFT theoretical calculations revealed that all the compounds are not planar, as illustrated in Figure 3.

### Biological Evaluation

#### *In*-*vitro* Antibacterial Activity

The synthesized compounds were screened to determine their *in vitro* antibacterial activity against gram positive bacteria (*Staphylococcus aureus, Streptococcus pyogenes*) and gram-negative bacterial strains (*Escherichia coli, Pseudomonas aeruginosa*) by agar diffusion method with different concentrations of the seleno-pyridones derivatives, as shown in Table 1. Ciprofloxacin and gentamicin were used as reference standard drugs (81 and 86% inhibition, respectively). All the tested synthetic compounds manifested moderate to excellent antibacterial activity against the bacterial strains. In general, as the concentration of seleno-pyridones molecules increases, the antibacterial activity also increases. Study results disclosed that compounds **9b**, **12b**, **14e**, **14f**, and **16d** at 400 μm/ml concentration showed moderate activity toward *S. aureus* with 53.8, 54.3, 58.5, 58.9, and 57.8% inhibition, whereas **9b**, **12b**, **14e**, **14f**, and **16d** exhibited modest activity against *S. pyogenes* with 52.6, 54.6, 61.3, 64.5, and 60.6% inhibition, respectively. Furthermore, synthesized compounds revealed maximum activity against *E. coli* and *P. aeruginosa*. Among the tested compounds, **9b**, **12b**, **14e**, **14f**, and **16d** at 400 μm/ml concentration were the most effective against *E. coli* with 72,4, 76.6, 70.3, 78.4, 71.6% inhibition, respectively. Likewise, compounds **9b**, **12b**, **14e**, **14f**, and **16d** exhibited remarkable activity against *P. aeruginosa* with 72.8, 78.4, 72.6, 77.4, 74.7% inhibition, respectively. This indicates the potential impact of the new selanone series against the tested bacterial strains.

**Table 1:** Antibacterial activity of the synthesized compounds at 400 μm/ml with the percentage inhibition against some bacterial strains.

**Compounds**	***S. aureus***	***S. pyogenes***	***E. coli***	***P. aeruginosa***
	**± SD**	**± SD**	**± SD**	**± SD**
**8b**	39.6 ± 0.05	37.6 ± 0.03	40.6 ± 0.01	41.9 ± 0.09
**9b**	53.8 ± 0.02	52.6 ± 0.1	73.7 ± 0.05	72.8 ± 0.09
**10b**	48.5 ± 0.09	45.9 ± 0.05	58.9 ± 0.13	56.3 ± 0.09
**12a**	43.6 ± 0.1	42.7 ± 0.06	53.8 ± 0.05	59.1 ± 0.17
**12b**	54.3 ± 0.05	54.6 ± 0.21	76.6 ± 0.1	78.5 ± 0.13
**12c**	42.7 ± 0.09	43.7 ± 0.14	56.5 ± 0.1	59.8 ± 0.15
**14d**	51.2 ± 0.09	56.2 ± 0.15	61.9 ± 0.1	63.6 ± 0.1
**14e**	58.5 ± 0.05	61.3 ± 0.05	70.3 ± 0.05	72.6 ± 0.05
**14f**	58.9 ± 0.05	64.5 ± 0.05	78.4 ± 0.05	77.3 ± 0.05
**16d**	57.8 ± 0.05	60.6 ± 0.05	71.6 ± 0.05	74.7 ± 0.05
**Ciprofloxacin: 89%**			**Gentamicin: 85%**	

#### *In*-*vitro* Antifungal Activity

Similarly, an *in vitro* assay of selenopyridones were performed to appraise their antifungal activity against *Candida albicans, Aspergillus niger*, and *Aspergillus clavatus* using agar diffusion method, where Griseofulvin was utilized as a reference drug. The obtaining activity performance is given in Table 2, and it is apparent that the maximum antifungal influence was achieved using higher concentrations of the tested compounds. Case in point, compounds **9b**, **12b**, **14f**, and **16d**, 400 μm/ml, manifested prominent activity against *C. albicans* with 77.1, 72.1, 78.1, and 78.6% inhibition, respectively. Meanwhile, compounds **9b**, **12b**, **14e**, **14f**, and **16d** displayed excellent activity against *A. niger* with 71.1, 72.4, 70.1, 77.4, and 71.3% inhibition, respectively. Moreover, compounds **9b**, **12b**, **14e**, **14f**, and **16d** were found to have a notable impact against *A. clavatus* with inhibition percentage of 68.5, 68.4, 63.9, 74.3, and 68.3, respectively.

**Table 2:** Antifungal activity of the synthesized compounds at 400 μm/ml with the percentage inhibition against fungal strains.

**Compound number**	***Candida albicans***	***Aspergillus niger***	***Aspergillus clavatus***
**8b**	58.6	61.8	56.1
**9b**	77.1	71.1	68.5
**10b**	63.2	62.1	57.9
**12a**	61.9	61.6	61.2
**12b**	72.1	72.4	68.4
**12c**	57.3	63.5	59.7
**14d**	69.5	61.9	62.7
**14e**	69.9	70.1	63.9
**14f**	78.1	77.4	74.3
**16d**	78.6	71.1	68.3
**Griseofulvin**	**88%**	**86%**	**90%**

### Molecular Docking

The docking studies against 1kzn were subjected to our selenium compound and the well-known antibacterial drug “gentamicin.” The 1kzn is a code for the 24 kDa gyrase fragment; DNA gyrase is a main protein involving in replication and transcription of bacterial circular DNA. Many antibacterial drugs are known to target DNA gyrase, inducing bacterial death (Lafitte et al., 2002). In term of the docking study, our compound divided into two groups: Group (A) [**9b, 12b, 14e, 14f**, and **16d**] which have high antibacterial activity and have score varies from (−6.47 to −7.41) which is reasonably less than the score achieved by the gentamicin (−8.79). Group (B) [**8b, 10b, 12a, 12c**, and **14d**] which have moderate antibacterial activity and have score varies from (−5.87 to 6.55).

**Figure 4:**
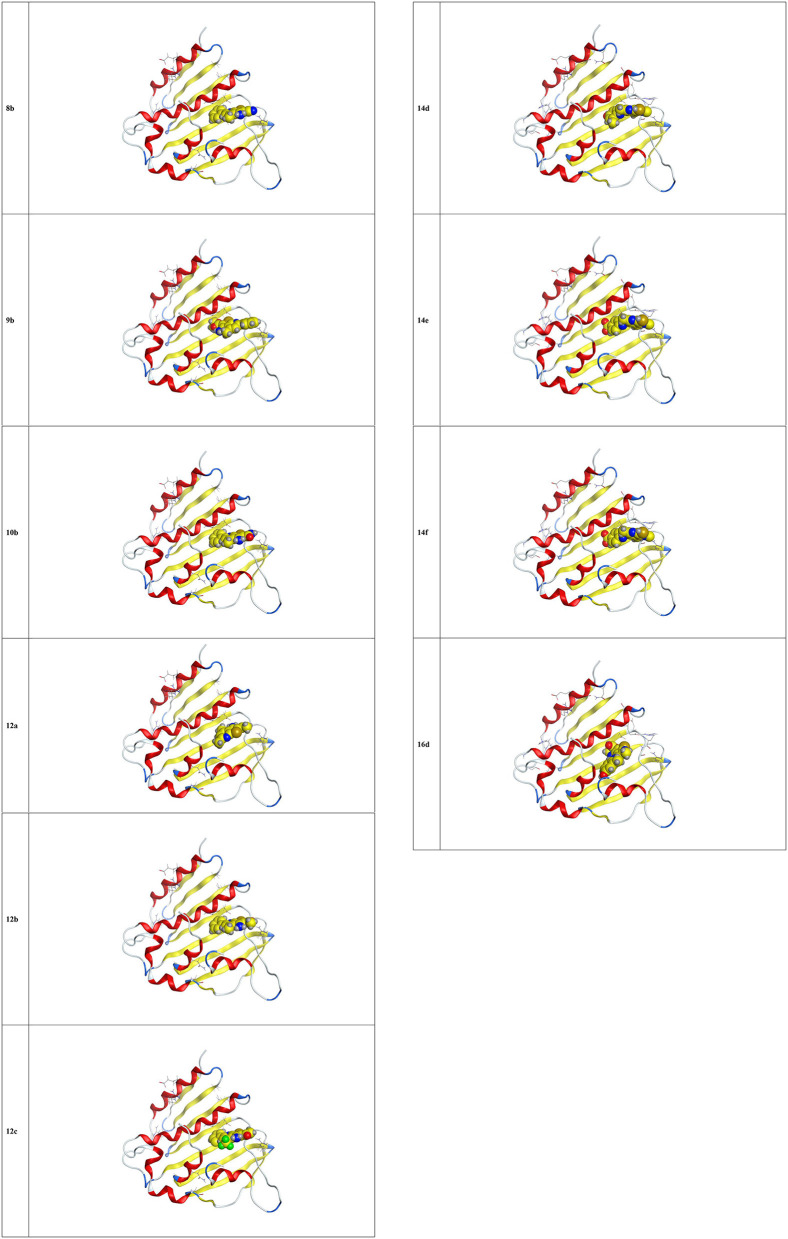
3D interaction of some selenium compounds with 1kzn protein.

**Figure 5:**
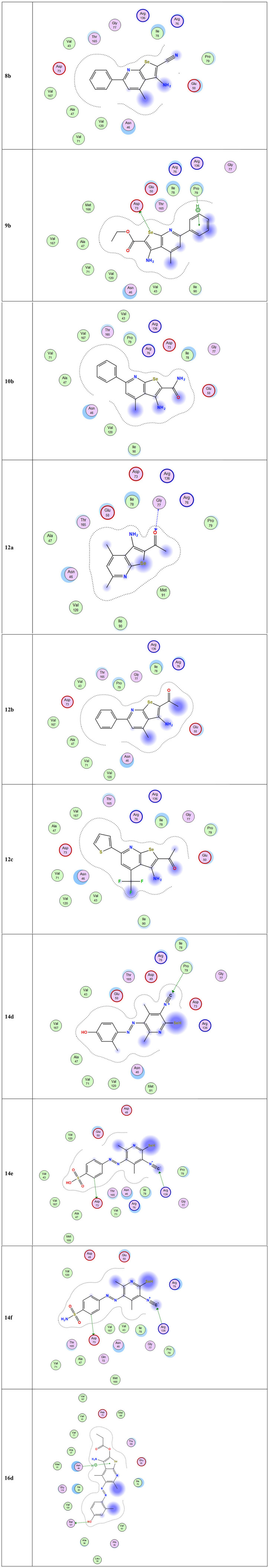
3D interaction of some selenium compound with 1kzn protein.

As apparent from the docking studies, Tables 3–5 and Figures 4, 5, all selenium compounds interact almost in the same site in the protein and hence our compound is expected to have antibacterial effect close from that seen by the gentamicin which is in agreement of what we observed in the experimental data.

**Table 3:** Docking score and energies of some selenium compounds with 1KZN protein.

**Compound**	**S**	**rmsd_refine**	**E_conf**	**E_place**	**E_score1**	**E_refine**	**E_score2**
**8b**	−6.35	1.09	9.32	−59.36	−9.06	−34.62	−6.35
	−5.95	1.29	9.84	−55.13	−8.53	−31.91	−5.95
	−5.92	0.75	9.89	−53.28	−8.58	−32.34	−5.92
	−5.91	0.79	13.3	−58.44	−8.94	−26.44	−5.91
	−5.82	2.66	9.72	−48.53	−9.99	−31.63	−5.82
**9b**	−7.17	2.31	−6.16	−35.28	−8.03	−41.03	−7.17
	−6.75	1.66	−8.99	−51.97	−10.67	−37.41	−6.75
	−6.63	1.93	−6.64	−40.55	−8.39	−29.77	−6.63
	−6.54	2.62	−6.51	−61.98	−10.22	−34.24	−6.54
	−6.52	1.22	−0.41	−70.49	−10.02	−36.96	−6.52
**10b**	−6.46	1.77	−52.5	−53.68	−8.94	−34.44	−6.46
	−6.39	1.85	−53.07	−50.09	−9.3	−35.92	−6.39
	−6.2	1.03	−52.71	−46.61	−10.6	−34.24	−6.2
	−6.12	1.9	−52.33	−47.65	−8.69	−34.89	−6.12
	−6.12	1.17	−52.42	−48.67	−9.48	−34.6	−6.12
**12a**	−5.87	0.88	−36.6	−62.42	−8.53	−23.49	−5.87
	−5.85	1.81	−33.32	−48.43	−8.96	−27.29	−5.85
	−5.8	1.41	−38	−41.74	−9.01	−28.17	−5.8
	−5.71	2.46	−39.21	−37.82	−8.94	−30.1	−5.71
	−5.64	1.35	−37.18	−45.03	−8.28	−25.45	−5.64
**12b**	−6.47	1.87	−7.36	−50.89	−8.91	−36.06	−6.47
	−6.4	1.37	−7.85	−50.7	−10.12	−35.66	−6.4
	−6.26	1.61	−7.68	−54.78	−8.87	−31.59	−6.26
	−6.26	1.17	−5.59	−60.43	−9.48	−27.16	−6.26
	−6.17	2.64	−7.68	−52.2	−8.5	−31.51	−6.17
**12c**	−6.55	2.12	18.12	−48.59	−10.7	−34.72	−6.55
	−6.51	1.59	17.98	−49.64	−9.83	−35.5	−6.51
	−6.34	0.95	21.22	−38.49	−8.71	−28.57	−6.34
	−6.27	1.22	21.21	−37.43	−8.43	−28.26	−6.27
	−6.27	2.38	18.97	−56.83	−10.65	−34.12	−6.27
**14d**	−6.71	0.9	18.91	−89.7	−10.5	−37.68	−6.71
	−6.71	1.18	21.59	−73.07	−9.73	−29.72	−6.71
	−6.5	1.33	22.82	−58.83	−10.39	−31.14	−6.5
	−6.42	1.74	21.31	−86.32	−9.81	−32.09	−6.42
	−6.33	2.7	20.78	−68.25	−10.03	−32.86	−6.33
**14e**	−7.05	1.04	41.45	−78.64	−10.89	−38.42	−7.05
	−7.01	1.91	43.89	−58.12	−9.55	−39.32	−7.01
	−6.96	1.27	47.61	−81.57	−9.77	−31.66	−6.96
	−6.96	1.46	41.44	−73.85	−9.52	−36.41	−6.96
	−6.89	1.43	40.92	−83.91	−10.42	−38.68	−6.89
**14f**	−6.97	1.22	9.78	−87.72	−10.81	−34.72	−6.97
	−6.83	0.97	9.68	−84.16	−9.49	−33.2	−6.83
	−6.73	1.66	7.83	−74.89	−9.59	−39.16	−6.73
	−6.59	2.04	12.32	−77.3	−9.7	−33.77	−6.59
	−6.58	0.77	12.15	−86.36	−9.4	−33.96	−6.58
**16d**	−8.48	1.48	59.62	−75.73	−12.1	−44.92	−8.48
	−8.22	1.59	51	−84.08	−11.21	−41.06	−8.22
	−7.4	1.13	50.21	−85.04	−10.48	−36.34	−7.4
	−7.27	1.16	46.64	−89.67	−11.04	−38.25	−7.27
	−7.16	2.02	52.57	−103.84	−10.33	−38.01	−7.16
Gentamycin	−8.79	1.8	204.44	−85.38	−11.96	−43.18	−8.79

*The colored provided reflects the strength of binding between compounds and protein*.

**Table 4:** Docking interaction of some selenium compounds with 1KZN protein.

**Compound**	**Ligand**	**Receptor**	**Interaction**	**Distance E**	**(kcal/mol)**
**8b**	No measurable interaction observed				
**9b**	Se 14	OD1 ASP 73 (A)	H-donor	3.49	−5.4
	6-ring	CD PRO 79 (A)	pi-H	3.85	−0.5
**10b**	No measurable interaction observed				
**12a**	O 16	N GLY 77 (A)	H-acceptor	2.90	−0.5
**12b**	No measurable interaction observed				
**12c**	No measurable interaction observed				
**14d**	C 35	CD PRO 79 (A)	H-acceptor	3.88	−0.6
**14e**	C 22	OD1 ASP 73 (A)	H-donor	3.24	−0.6
	C 30	NH1 ARG 136 (A)	H-acceptor	3.29	−4.2
**14f**	C 22	OD1 ASP 73 (A)	H-donor	3.25	−0.6
	C 30	NH1 ARG 136 (A)	H-acceptor	3.28	−4.7
16	O 36	OG SER 121 (A)	H-donor	3.15	−0.9
	5-ring	CB ASN 46 (A)	pi-H	4.30	−0.5

**Table 5:** Docking score of the synthesized compounds against bacterial strains.

**Compounds**	***S. aureus***	***S. pyogenes***	***E. coli***	***P. aeruginosa***	**Docking score**
**8b**	39.6	37.6	40.6	41.9	
**9b**	53.8	52.6	73.7	72.8	
**10b**	48.5	45.9	58.9	56.3	
**12a**	43.6	42.7	53.8	59.1	
**12b**	54.3	54.6	76.6	78.5	
**12c**	42.7	43.7	56.5	59.8	
**14d**	51.2	56.2	61.9	63.6	−6.71
**14e**	58.5	61.3	70.3	72.6	−7.01
**14f**	58.9	64.5	78.4	77.3	−6.97
**16d**	57.8	60.6	71.6	74.7	−8.48
**Ciprofloxacin:**	**89%**		**Gentamicin:**	**85%**	

### *In-silico* ADME Study

The predicted pharmacokinetic/Molinspiration properties (Lipinski et al., 1997; Molinspiration, 2011; Singh et al., 2017) of the new series, **8b, 9b, 10b, 12a-c, 14d-f**, and **16d** are given in Tables 6, 7. With the help of Molinspiration virtual screening, most of the synthesized compounds showed promising bioactivity as implied from docking parameters in Tables 6, 7, which indicates the drug likeness properties against kinase inhibitor, protease and enzyme inhibitors. On the other hand, compounds 8b, 10b, and 12b-c showed no activity since the activities were out the overlapped area between the enzyme inhibitors and drug-likeness molecules.

**Table 6:** Physicochemical properties of the synthesized compounds.

**Compound**	**miLogP**	**TPSA**	**natoms**	**MW**	**nON**	**nOHNH**	**nviolations**	**nrotb**	**Volume**
**8b**	3.43	62.71	19	312.23	3	2	0	1	236.07
**9b**	3.89	65.22	22	359.29	4	2	0	4	280.54
**10b**	2.5	82.01	20	330.25	4	4	0	2	249.48
**12a**	2.13	55.99	15	267.19	3	2	0	1	199.91
**12b**	3.58	55.99	20	329.26	3	2	0	2	254.76
**12c**	3.81	55.99	22	389.26	3	2	0	3	260.20
**14d**	−2.82	62.21	21	345.26	5	1	0	2	272.39
**14e**	0.78	115.78	23	395.30	7	1	0	3	282.03
**14f**	2.48	121.58	23	394.32	7	2	0	3	285.30
**16d**	4.94	110.18	27	431.35	7	3	0	5	340.79

**Table 7:** Physicochemical Molinspiration bioactivity score.

**Compound**	**GPCR ligand**	**Ion channel modulator**	**Kinase inhibitor**	**Nuclear receptor ligand**	**Protease inhibitor**	**Enzyme inhibitor**
**8b**	0.04	−0.07	0.59	−0.18	−0.25	0.16
**9b**	0.00	−0.12	0.37	−0.12	−0.23	0.03
**10b**	0.08	−0.10	0.59	−0.27	−0.15	0.16
**12a**	−0.28	−0.34	−0.00	−0.82	−0.61	−0.12
**12b**	0.02	−0.11	0.41	−0.25	−0.25	0.11
**12c**	0.00	−0.19	0.42	−0.10	−0.27	0.04
**14d**	−0.20	−0.27	−0.13	−0.51	−0.49	0.07
**14e**	0.01	−0.13	−0.04	−0.64	0.06	0.06
**14f**	−0.21	−0.33	−0.06	−0.62	−0.09	0.04
**16d**	0.08	−0.15	0.21	0.24	−0.15	0.11

### Chemical Reactivity Descriptors

Interestingly, the computational investigation by DFT is an excellent tool in designing of new materials and attracts attentions of many researchers in recent reports in illustration of the biological activities (Hagar et al., 2020a; Almehmadi et al., 2021; Mohammed et al., 2021; Parveen et al., 2021). Recently, the frontier orbitals, the highest occupied molecular orbital (HOMO), the lowest unoccupied molecular orbital (LUMO) and their energy gaps have been used to prove numerous chemical reactivity forms of the biological characters. Moreover, several biological activities such as antifungal (Joshi et al., 2018, 2020), anticancer (Kumar et al., 2018; Hagar et al., 2019; Khodair et al., 2020), antimicrobial (Grover et al., 2000; Malhotra et al., 2017; Kumer et al., 2019), cytotoxic (Aljuhani et al., 2018; Da Costa et al., 2018; Rachedi et al., 2019) activities and a new-drug-design field (Lewis, 2003) could be investigated in terms of the relationship with the energy of the FMOs.

Diverse important factors, such as the potential of the electronic ionization (I = –E_HOMO_) and the electron affinity of the LUMO (A = –E_LUMO_) could be estimated from the FMOs. Furthermore, the FMOs is an excellent tool to appraise various chemical reactivity descriptors, such as softness (δ), global hardness (η), electronegativity (χ) (an indicator for the electronic acceptance ability of the compound i.e., Lewis acidity), and electrophilicity (ω) (an appraisal for the lower energy of electronic transition), which could be calculated as followed (Ortega et al., 2020) and shown in Table 8.

(1) $$\begin{array}{r} \chi =-\frac{1}{2}\left(E_{HOMO}+E_{LUMO}\right) \end{array}$$

(2) $$\begin{array}{r} \eta =-\frac{1}{2}\left(E_{HOMO}-E_{LUMO}\right) \end{array}$$

(3) $$\begin{array}{r} \delta =\frac{1}{\eta } \end{array}$$

(4) $$\begin{array}{r} \omega =\frac{\chi^{2}}{2\eta } \end{array}$$

Recently, the energy level and the energy gap of the frontier molecular orbitals (HOMO and LUMO) were reported to influence the binding affinity of the compounds and to direct the interactive mode with the receptor proteins.

**Table 8:** Chemical reactivity descriptors and dipole moment (μ, Debye) of investigated compounds.

**Compound**	**HOMO**	**LUMO**	**ΔE**	**χ**	**η**	**δ**	**ω**	**I**	**A**	**μ**
**8b**	−5.63	−1.94	3.69	3.79	1.84	0.54	3.88	1.94	5.63	7.5510
**9d**	−5.38	−1.85	3.53	3.61	1.76	0.57	3.70	1.85	5.38	4.1009
**10b**	−5.32	−1.74	3.58	3.53	1.79	0.56	3.48	1.74	5.32	3.1917
**12a**	−5.50	−1.95	3.55	3.73	1.78	0.56	3.91	1.95	5.50	4.4184
**12b**	−5.53	−2.22	3.31	3.88	1.65	0.60	4.55	2.22	5.53	4.2244
**12c**	−5.83	−2.94	2.89	4.39	1.45	0.69	6.66	2.94	5.83	4.2920
**14d**	−5.92	−1.84	4.08	3.88	2.04	0.49	3.68	1.84	5.92	6.4304
**14e**	−6.55	−3.04	3.51	4.80	1.76	0.57	6.56	3.04	6.55	3.9722
**14f**	−6.49	−2.66	3.83	4.57	1.92	0.52	5.46	2.66	6.49	2.3212
**16d**	−5.64	−1.87	3.77	3.75	1.88	0.53	3.74	1.87	5.64	7.3721

**Figure 6:**
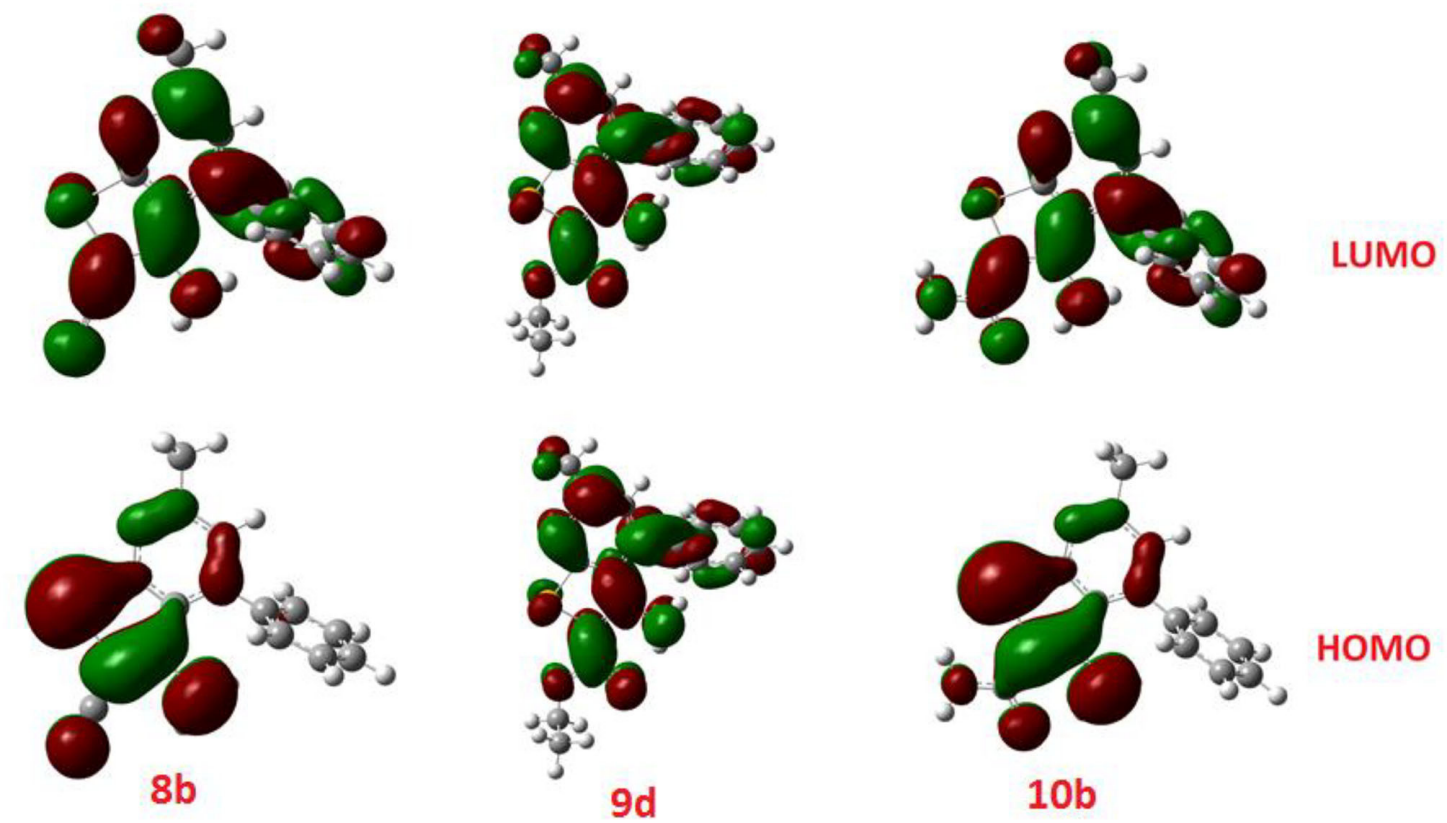
The calculated ground state isodensity surface plots of the FMOs for **8b, 9d**, and **10b**.

The calculated ground state isodensity surface plots of the FMOs energy levels and the energy gap of selenopheno[2, 3-b]pyridines (**8b**, **9d**, and **10b**) are illustrated in Figure 6. It is clear that the energy levels and the energy gap of the FMOs for these compounds have no significant effect to the attached substituent of the selenopheno ring. This result could be attributed to the lack of extra conjugation with the attached groups. However, the lower energy gap of **9d** in comparison with the others could illustrate its lower binding energy.

**Figure 7:**
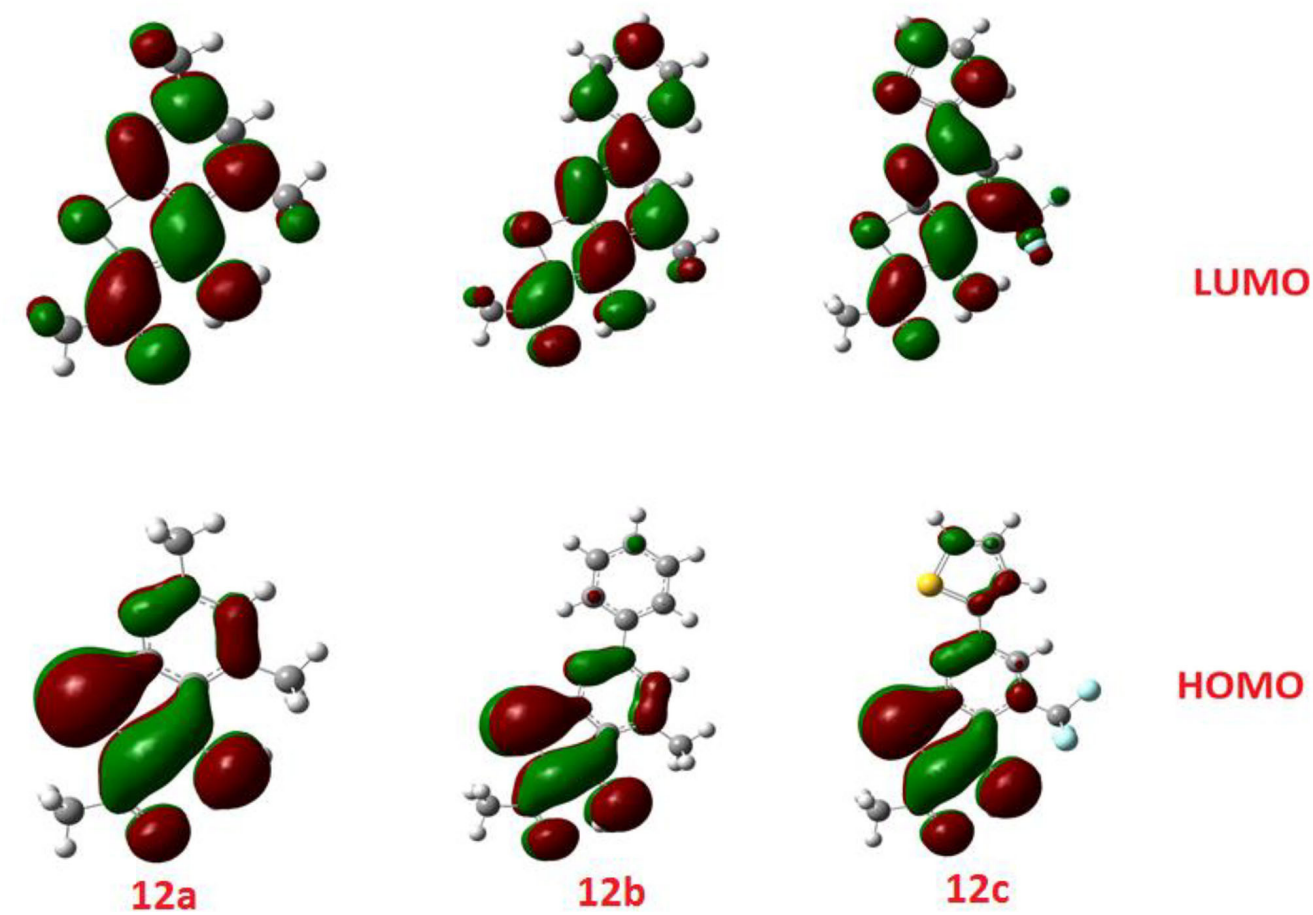
The calculated ground state isodensity surface plots for FMOs for the investigated compounds **12(a-c)**.

The comparison of the FMO levels of the prepared compounds **12a-c** of the attached aromatic rings to the pyridine moiety is a good explanation for the higher appraisal molecular docking score of compound **12c** with respect to the other compounds. The high lying HOMO of **12c** permits a higher ability to donate electrons to the receptors either in the anticancer or antimicrobial cell/protein. The high lyophobicity of **12b** along with the affected chemical descriptors, higher softness δ = 0.60, higher basicity χ = 3.88 as well as higher ω = 3.88 along with the level and the gap of the FMOs is an illustration of the molecular docking results as well as its biological activity, Figure 7.

**Figure 8:**
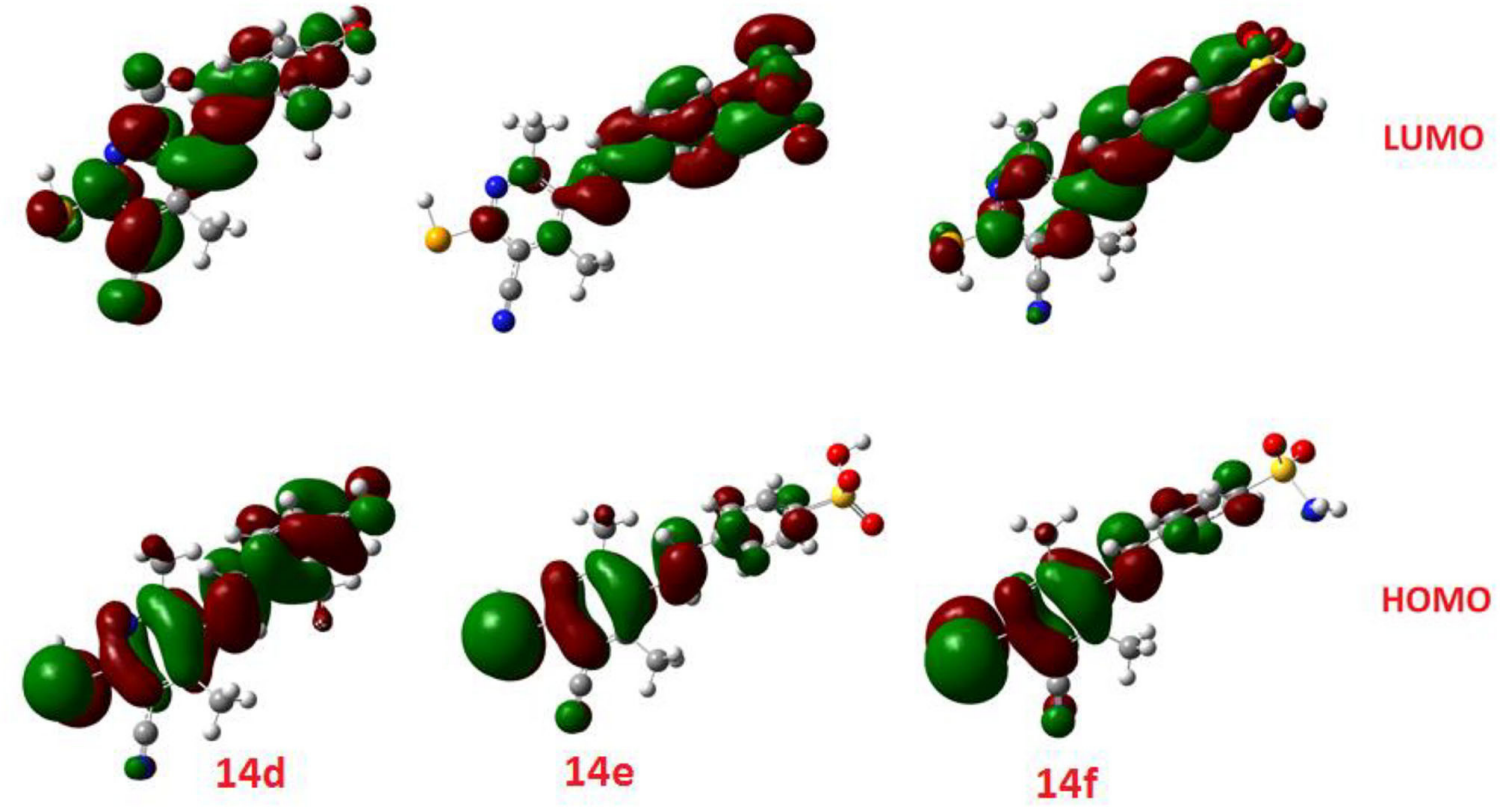
The calculated ground state isodensity surface plots for FMOs for the investigated compounds **14(d-f)**.

Similarly, the FOMs energy levels and their energy difference of hydroselenonicotinonitrile **14d-e** were also calculated. It is obvious from Figure 8 that compound **14e** with benzenesulfonic acid moiety has a lower energy gap than that of other derivatives. However, the lower hydrophobicity, higher topological polar surface area, lower dipole moment, high H-bonding acceptor and donor, and *in*-*silico* absorption percentage of **14f** explicates its highest biological activity.

### Molecular Electrostatic Potential

To verify the evidence regarding the investigated reactivity of the compounds as enzyme inhibitors, the molecular electrostatic potential (MEP) is an important parameter to be predicted. Since the MEP defines the molecular size and shape of the positive, negative, and the neutral electrostatic potentials, the MEP can be an indicator for expecting physiochemical property relationships with the molecular structure. Furthermore, the molecular electrostatic potential is a useful tool in the prediction of the susceptibility of the studied compounds toward electrophiles and nucleophiles.

**Figure 9:**
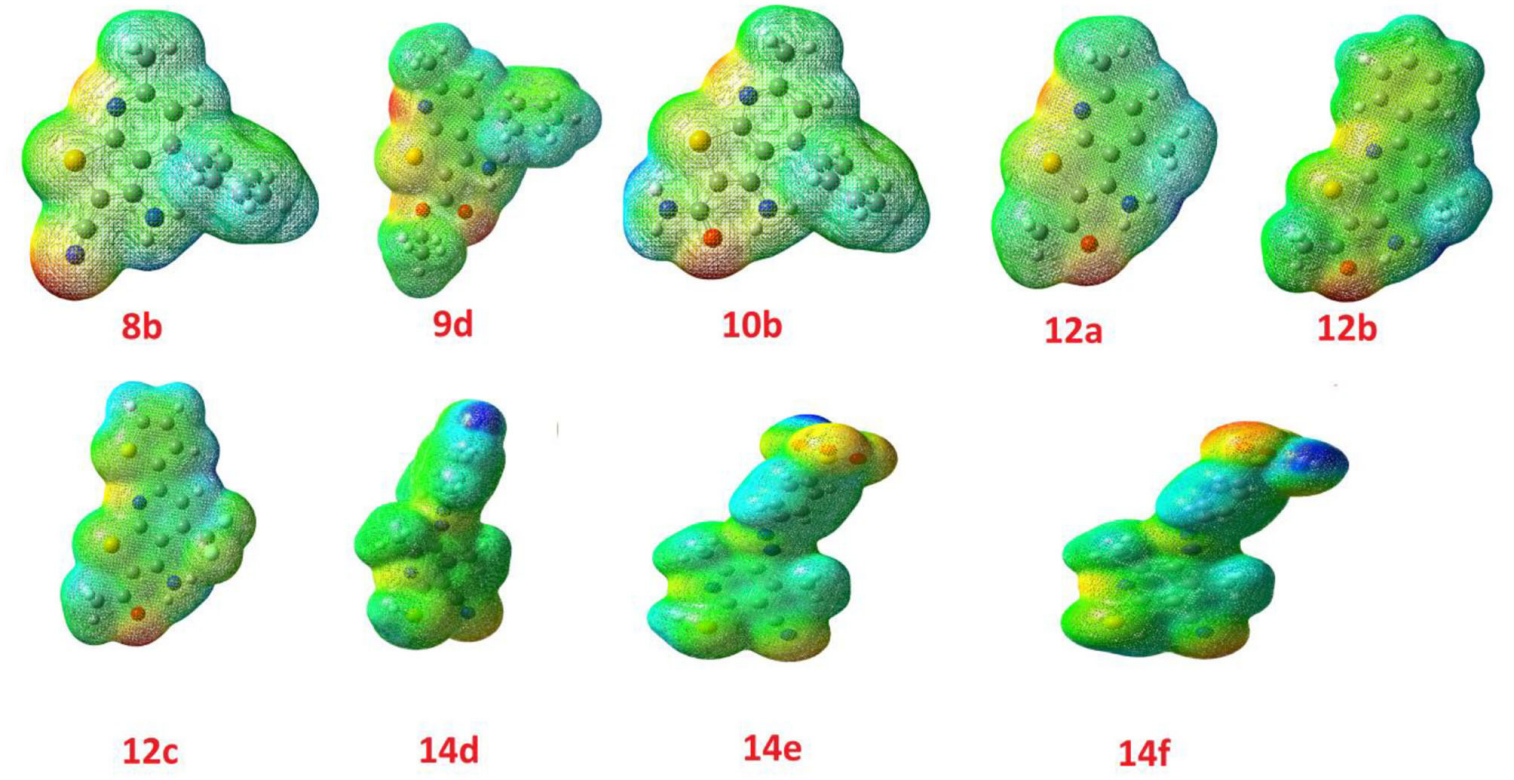
Molecular electrostatic potentials (MEP) of for the investigated compounds.

The molecular electrostatic potential was calculated by the same method under the same base sets and given in Figure 9. In the MEP, the higher negative region is the favored sites for electrophilic attack demonstrated in the red color, Figure 3. An electrophile attack is attracted to the negatively charged sites and vice versa for the blue regions. Additionally, it is evident that the molecular size, shape, and orientation of the negative, positive and neutral electrostatic potential are varied according to the electronic nature of the compounds as well as the electronegativity of their attached groups. The difference in the electrostatic potential mapping of the compounds could be the reasoning behind the extent of the binding affinity of the studied compounds to activate the receptor's sites.

## Experimental

### Chemistry

#### General

Melting point determination was done using open capillary tubes on an electrical melting point apparatus. Bruker spectrospin DPX-400MHz was used to record the ^1^H NMR and ^13^C NMR spectra. Chemical shift (δ) values were stated in parts per million (ppm) using internal standard tetramethylsilane. The D_2_O exchange confirmed the exchangeable protons (OH and NH). LC–MS/MS (PerkinElmer) was used to record the mass spectra, presented as *m/z*. Elemental analyses were achieved by using PerkinElmer 240 analyzer. The purity of synthesized compounds as well as progress of reaction were assessed by ascending thin layer chromatography (TLC) (silica gel G) by using methanol/chloroform (9:1 v/v) and methylene chloride/chloroform (4:1 v/v) combination as solvent system.

#### General Procedure for the Synthesis of 2-seleno-4,6-disubstitutednicotinonitrile Derivatives (4a-c)

A solution of 3-Cyano-2(1*H*)-pyridones (**1a-c**) (10 mmol), and phosphrous oxychloride (POCl_3_) (30 mmol), was irradiated with microwave for 7 min, then NaSeH (1 g, 12 mmol) were mixed with the resulting solution in a HP-500 Plus process vessel. The process vessel was capped properly and exposed by microwaves under suitable pressurized conditions (17.2 bar, 220°C) for 3–5 min. After irradiation, reaction mixture was cooled and added in crushed ice. The mixture was sonicated for 1 h and filtered in a sintered glass followed by solvent evaporation in *vacuo*, and the resultant product was recrystallized from mixture of EtOH/DMF to afford (Scheme 1) (**4a-c**).

#### General Procedure for the Synthesis of Selenium Derivatives 11a-c, 12a-c, 5b, 6b, 7b, 8b, 9b, and 10b

- A mixture of 4(a-c) (10 mmol), chloroacetone (10 mmol) and sodium acetate (10 mol) was charged in a process vessel (HP-500 Plus) for 3–5 min. The vessel was capped properly and irradiated by microwaves under suitable pressurized conditions (18 bar, 250°C). The reaction mixture was cooled in iced water/ethanol (100 ml) and recrystallized from ethanol to give compounds 11(a-c). Furthermore, compound 11(a-c) (10 mmol) was mixed with ethanol and sodium acetate and then charged in a process vessel (HP-500 Plus) under the same conditions as mentioned above to give compounds 12(a-c).
- Mixture of ClCH_2_X and sodium acetate 10 (mmol) was processed by the same method as mentioned in section General Procedure for the Synthesis of 2-Seleno-4,6-Disubstitutednicotinonitrile Derivatives (Scheme 1) (4a-c) to give compounds 5(b), 6(b), and 7(b), where X was CN, COOEt and CONH_2_, respectively.
- Compounds 5(b), 6(b), and 7(b) were refluxed in glacial acetic acid and/or irradiated with microwave for 2 min to give compounds 8(b), 9(b), and 10(b), respectively.

#### General Procedure for the Synthesis of Selenium Derivatives 14d-f

Compounds (14d-f) were synthesized by adopting same procedure as given in section General Procedure for the Synthesis of 2-Seleno-4,6-Disubstitutednicotinonitrile Derivatives (Scheme 1) (4a-c) by taking (1d-f) as starting material.

#### General Procedure for the Synthesis of Selenium Derivatives 16d

A mixture of 14(d) (10 mmol), ClCH_2_COOEt and sodium acetate 10 (mmol) was treated by same procedure as mentioned in section General Procedure for the Synthesis of Selenium Derivatives 11a-c, 12a-c, 5b, 6b, 7b, 8b, 9b, and 10b (i) to give compound 15(d). Furthermore, compound 15(d) reacted with glacial acetic acid (2 ml) and microwaved for 2 min to give compound 16(d), purified and recrystallized from pure ethanol.

##### 4,6-dimethyl-2-selenoxo-1,2-dihydropyridine-3-carbonitrile (4a)

Solid, brownish; yield: 94%; m.p. (°C): 461.3; IR (KBr) ν cm^−1^: 2,222 (C–N), 3,210 (N–H); ^1^H NMR (CDCl_3_): (δ ppm) 2.1 (s, -CH_3_, 3H), 2.04 (s, -CH_3_, 3H), 6.7 (s, -CH, Ar-H); ^13^C NMR (CDCl_3_): (δ ppm) 19.8 (s, C, CH_3_), 20.1 (s, C, CH_3_), 118.3 (s, C, CN), 153 (s, C2, Ar), 96.5 (s, C3, Ar), 156.7 (s, C4, Ar), 115.4 (s, C5, Ar), 163.2 (s, C6, Ar); ESI MS (*m/z*): 212.9 [M+H]^+^. Anal. calcd. For C_8_H_8_N_2_Se: C: 45.51, H: 3.82, N: 13.27, Se: 37.40; Found: C: 45.46, H: 3.81, N: 13.30%.

##### 2-(2-oxopropylselanyl)-4-(thiophen-2-yl)-6-(trifluoromethyl)nicotinonitrile (4b)

Solid, dark brown; yield: 91%; m.p. (°C): 626.5; IR (KBr) ν cm^−1^: 2,225 (C–N), 1,720 (C=O); ^1^H NMR (CDCl_3_): (δ ppm) 1.7 (s, -CH_3_, 3H), 2.2 (s, -CH_2_, 2H), 7.21 (s, -CH, Ar-H), 7.6 (dd, H, C1 thienyl), 7.4 (dd, H, C2 thienyl), 7.3 (dd, H, C3 thienyl); ^13^C NMR (CDCl_3_): (δ ppm) 28.1 (s, C, CH_3_), 36.3 (s, C, -CH_2_), 206.1 (s, C, CO), 118.3 (s, C, CN) 154 (s, C2, Ar), 97.2 (s, C3, Ar), 159.7 (s, C4, Ar), 113.4 (s, C5, Ar), 154.2 (s, C6, Ar), 217.1 (m, -CF_3_), 125.3 (s, C2 thienyl), 127.1 (s, C3 thienyl), 125.2 (s, C4 thienyl), 135.4 (s, C5, thienyl); ESI MS (*m/z*): 390 [M+H]^+^. Anal. calcd. For C_14_H_9_F_3_N_2_OSSe: C: 43.20, H: 2.33, N: 7.20, F: 14.64, S: 8.24, Se: 20.28; Found: C: 43.31, H: 2.33, N: 7.21, F: 14.68, S: 8.23%.

##### 4-methyl-2-(2-oxopropylselanyl)-6-phenylnicotinonitrile (4c)

Solid, brown; yield: 92%; m.p. (°C): 818.5; IR (KBr) ν cm^−1^: 1,650 (C=O), 2,220 (C–N); ^1^H NMR (CDCl_3_): (δ ppm) 1.56 (s, -CH_3_), 1.77 (s, CH_3_), 2.3 (s, -CH_2_), 7.6 (s, CH, Ar), 7.8 (m, 4H, ph); ^13^C NMR (CDCl_3_): (δ ppm) 28.1 (s, C, CH_3_), 36.3 (s, C, -CH_2_), 206.1 (s, C, CO), 118.3 (s, C, CN), 154 (s, C2, Ar), 97.2 (s, C3, Ar), 156.7 (s, C4, Ar), 123.4 (s, C5, Ar), 166.2 (s, C6, Ar), 141.1 (s, C1, ph), 127.5 (s, s, C2, C6, ph), 129.4 (s, s, C3, C5), 125 (s, C6); ESI MS (*m/z*): 331 [M+H]^+^. Anal. calcd. for C_16_H_14_N_2_OSe: C: 58.37, H: 4.29, N: 8.51, Se: 23.98; Found: C: 58.42, H: 4.28, N: 8.53%.

##### (E)-5-((4-hydroxy-2-methylphenyl)diazenyl)-4,6-dimethyl-2-selenoxo-1,2-dihydropyridine-3-carbonitrile (14d)

Solid, brownish; yield: 94%; m.p. (°C): 719.2; IR (KBr) ν cm^−1^: 3,550 (O–H), 2,225 (C–N), 3,235 (N–H); ^1^H NMR (CDCl_3_): (δ ppm) 1.72 (s, CH_3_, 3H), 1.83 (s, CH_3_, 3H), 2.04 (s, CH_3_, 3H), 4.32 (s, OH), 4.23 (s, NH), 7.2 (dd, 3H, Ar-H); ^13^C NMR (CDCl_3_): (δ ppm) 18.1 (s, CH_3_), 19.9 (s, CH_3_), 23.3 (s, CH CH_3_), 36.6 (s, CH, Ar), 85 (s, C3, Ar), 117.5 (s, CN), 161.0 (s, C4, Ar), 121 (s, C5, Ar), 125 (s, C6, Ar), 122 (s, C1, Ar), 139 (s, C2, Ar), 115 (s, C3, Ar), 155.5 (s, C4, Ar), 112 (s, C5, Ar), 129 (s, C6, Ar); ESI MS (*m/z*): 347 [M+H]^+^. Anal. calcd. for C_15_H_14_N_4_OSe: C: 52.18, H: 4.09, N: 16.23, Se: 22.87; Found: C: 52.04, H: 4.09, N: 16.19%.

##### (E)-4-((5-cyano-2,4-dimethyl-6-selenoxo-1,6-dihydropyridin-3-yl)diazenyl)benzenesulfonamide (14e)

Solid, Dark brown; yield: 93%; m.p. (°C): 683; IR (KBr) ν cm^−1^: 3,310 (N–H), 2,250 (C–N), 3,300, 3,400 NH_2_; ^1^H NMR (CDCl_3_): (δ ppm) 1.84 (s, CH_3_, 3H), 1.8 (s, CH_3_, 3H), 7.8 (dd, 4H, Ar-H), 4.2 (s, -CH, Ar), 2.8 (s, s, NH, NH_2_); ^13^C NMR (CDCl_3_): (δ ppm) 17.8 (s, CH_3_), 23 (s, CH_3_), 36.6 (s. CH, Ar), 83 (s, C3, Ar), 116.5 (s, CN), 160 (s, C4, Ar), 120 (s, C5, Ar), 125 (s, C6, Ar), 122 (s, C1, Ar), 130 (s, s, C2, C6, Ar), 126 (s, s, C3, C5, Ar), 144.5 (s, C4, Ar); ESI MS (*m/z*): 396.3 [M+H]^+^. Anal. calcd. for C_14_H_13_N_5_O_2_SSe: C: 42.64, H: 3.32, N: 17.76, S: 8.13, Se: 20.02; Found: C: 42.55, H: 3.32, N: 17.76, S: 8.12%.

##### (E)-4,6-dimethyl-2-selenoxo-5-((3,4,5-trimethoxyphenyl)diazenyl)-1,2-dihydropyridine-3-carbonitrile (14f)

Solid, brown; yield: 91%; m.p. (°C): 721.2; IR (KBr) ν cm^−1^: 3,210 (N–H), 2,223 (C–N); ^1^H NMR (CDCl_3_): (δ ppm) 1.7 (s, CH_3_, 3H), 1.84 (s, CH_3_, 3H), 3.14 (s, 3H, CH_3_), 6.45 (s, 2CH, Ar); ^13^C NMR (CDCl_3_): (δ ppm) 18.1 (s, CH_3_), 23.3 (s, CH_3_), 36.6 (s, CH, Ar), 85 (s, C3, Ar), 117.5 (s, CN), 161 (s, C4, Ar), 121 (s, C5, Ar), 125 (s, C6, Ar), 122 (s, C1, Ar), 107 (s, s, C2, C4, Ar), 147 (s, s, C3,C5, Ar), 132 (s, C6, Ar), 65.6 (t, 3CH_3_-Ar); ESI MS (*m/z*): 407 [M+H]^+^. Anal. calcd. for C_17_H_18_N_4_O_3_Se: C: 50.38, H: 4.48, N: 13.82, Se: 19.48; Found: C: 50.28, H: 4.49, N: 13.80%.

##### 1-(3-amino-4,6-dimethylselenopheno[2,3-b]pyridin-2-yl)ethanone (12a)

Solid, brownish; yield: 94%; m.p. (°C): 409.1; IR (KBr) ν cm^−1^: 1,650 (C=O), 3,300, 3,400 (NH_2_); ^1^H NMR (CDCl_3_): (δ ppm) 1.7 (s, CH_3_, 3H), 1.77 (s, CH_3_, 3H), 1.9 (s, 3H, CH_3_), 5.4 (s, 2H, NH_2_); ^13^C NMR (CDCl_3_): (δ ppm) 19.2 (s, 2 CH_3_), 159 (s, C2, Ar), 105 (s, C3, Ar), 147 (s, C4, Ar), 91.2 (s, C2, Ar), 126 (C6, Ar), 108 (s, C2, indenyl), 199 (s, CO), 138 (s, C3, indenyl); ESI MS (*m/z*): 269 [M+H]^+^. Anal. calcd. for C_11_H_12_N_2_OSe: C: 49.45, H: 4.53, N: 10.48, Se: 29.55; Found: C: 49.40, H: 4.53, N: 10.50%.

##### 1-(3-amino-4-(thiophen-2-yl)-6-(trifluoromethyl) selenopheno[2,3-b] pyridin-2-yl)ethanone (12b)

Solid, light brown; yield: 89%; m.p. (°C): 560; IR (KBr) ν cm^−1^: 1,645 (C=O), 3,300, 3,400 (NH_2_); ^1^H NMR (CDCl_3_): (δ ppm) 1.7 (s, -CH_3_, 3H), 5.1 (s, -NH_2_, 2H), 7.21 (s, -CH, Ar-H), 7.6 (m, 3H, C1 thienyl), 7.6 (s, CH, Ar); ^13^C NMR (CDCl_3_): (δ ppm) 28.5 (s, CH_3_), 198 (s, CO), 110 (s, C2 indenyl), 140 (s, C3 indenyl), 151 (s, C2, Ar), 103 (s, C3, Ar), 218 (m, CF_3_), 153 (s, C4, Ar), 92 (s, C5, Ar), 129 (s, C6, Ar), 125.3 (s, C2 thienyl), 127.1 (s, C3 thienyl), 125.2 (s, C4 thienyl), 135.4 (s, C5, thienyl); ESI MS (*m/z*): 390.9 [M+H]^+^. Anal. calcd. for C_14_H_9_F_3_N_2_OSSe: C: 43.20, H: 2.33, N: 7.20, F: 14.64, S: 8.24, Se: 20.28; Found: C: 43.16, H: 2.33, N: 7.21, F: 14.62, S: 8.24%.

##### 1-(3-amino-4-methyl-6-phenylselenopheno[2,3-b] pyridin-2-yl) ethanone (12c)

Solid, brown; yield: 94%; m.p. (°C): 492; IR (KBr) ν cm^−1^: 1,680 (C=O); 3,350, 3,455 (NH_2_); ^1^H NMR (CDCl_3_): (δ ppm) 1.5 (s, CH_3_), 1.7 (s, CH_3_), 5.2 (s, NH_2_), 6.5 (s, CH, Ar), 7.8 (m, 5H, Ar); ^13^C NMR (CDCl_3_): (δ ppm) 28.1 (s, C, CH_3_), 36.3 (s, C, -CH_2_), 206.1 (s, C, CO), 154 (s, C2, Ar), 97.2 (s, C3, Ar), 156.7 (s, C4, Ar), 123.4 (s, C5, Ar), 166.2 (s, C6, Ar), 141.1 (s, C1, ph), 127.5 (s, s, C2, C6, ph), 129.4 (s, s, C3, C5, ph), 108 (s, C2, indenyl), 139 (s, C3, indenyl); ESI MS (*m/z*): 331 [M+H]^+^. Anal. calcd. for C_16_H_14_N_2_OSe: C: 58.37, H: 4.29, N: 8.51, Se: 23.98; Found: C: 58.28, H: 4.29, N: 8.50%.

##### 3-amino-4-(thiophen-2-yl)-6-(trifluoromethyl)selenopheno [2,3-b]pyridine-2-carbonitrile (8b)

Solid, brownish; yield: 91%; m.p. (°C): 678; IR (KBr) ν cm^−1^: 3,350, 3,455 (NH_2_), 2,225 (C–N); ^1^H NMR (CDCl_3_): (δ ppm) 5.1 (s, NH_2_), 7.5 (m, 3CH, thienyl), 6.8 (s, CH, Ar); ^13^C NMR (CDCl_3_): (δ ppm) 77 (t, 4C, theinyl), 118 (s, CN), 89 (s,C2 indenyl), 143 (s, C3 indenyl), 151 (m, C2, Ar), 103 (s, C3, Ar), 217 (m, CF_3_), 163 (s, C4, Ar), 92 (s, C5, Ar), 129 (s, C6, Ar), 125.3 (s, C2 thienyl), 127.1 (s, C3 thienyl), 125.2 (s, C4 thienyl), 135.4 (s, C5, thienyl); ESI MS (*m/z*): 373.9 [M+H]^+^. Anal. calcd. for C_13_H_6_F_3_N_3_SSe: C: 41.95, H: 1.62, N: 11.29, F: 15.31, S: 8.61, Se: 21.21; Found: C: 42.0, H: 1.62, N: 11.30, F: 15.30, S: 8.63.

##### Ethyl 3-amino-4-(thiophen-2-yl)-6-(trifluoromethyl) selenopheno[2,3-b] pyridine-2-carboxylate (9b)

Solid, Dark brown; yield: 94%; m.p. (°C): 647.6; IR (KBr) ν cm^−1^: 3,200, 3,350 (NH_2_); ^1^H NMR (CDCl_3_): (δ ppm) 0.6 (t, 3H, CH_3_), 3.1 (q, 2H, CH2), 5.1 (s, 2H, NH_2_), 7.5–7.6 (m, 3H, theinyl); ^13^C NMR (CDCl_3_): (δ ppm) 13.5 (s, C, CH_3_), 60.8 (s, C, CH_2_), 77.5 (t, CH_3_, CH_2_), δ 77.5 (t, C, CF_3_), 217–220 (m, C, CF_3_), 151 (m, C2, Ar), 103 (s, C3, Ar), 217 (m, CF_3_), 163 (s, C4, Ar), 92 (s, C5, Ar), 129 (s, C6, Ar), 125.3 (s, C2 thienyl), 127.1 (s, C3 thienyl), 125.2 (s, C4 thienyl), 135.4 (s, C5, thienyl), 106.2 (s, C2 indenyl), 139 (s, C3 indenyl), 174(s CO); ESI MS (*m/z*): 420.9 [M+H]^+^. Anal. calcd. for C_15_H_11_F_3_N_2_O_2_SSe: C: 42.97, H: 2.64, N: 6.68, F: 13.59, S: 7.65, Se: 18.83; Found: C: 42.90, H: 2.64, N: 6.66, F: 13.55, S: 7.64.

##### 3-amino-4-(thiophen-2-yl)-6-(trifluoromethyl)selenopheno[2,3-b]pyridine-2-carboxamide (10b)

Solid, Dark brown; yield: 92%; m.p. (°C): 635; IR (KBr) ν cm^−1^: 3,226, 3,400 (2NH_2_), 1717 (C=O); ^1^H NMR (CDCl_3_): (δ ppm) 5.6 (s, 4H, 2NH_2_), 6.7 (s, H, CH, Ar), 7.2–7.5 (m, 3H, theinyl); ^13^C NMR (CDCl_3_): (δ ppm) 77.5 (t, C, CF_3_), 217–220 (m, C, CF_3_), 151 (m, C2, Ar), 103 (s, C3, Ar), 217 (m, CF_3_), 163 (s, C4, Ar), 92 (s, C5, Ar), 129 (s, C6, Ar), 125.3 (s, C2 thienyl), 127.1 (s, C3 thienyl), 125.2 (s, C4 thienyl), 135.4 (s, C5, thienyl), 106.2 (s, C2 indenyl), 139 (s, C3 indenyl), 169 (s, CO); ESI MS (*m/z*): 392 [M+H]^+^. Anal. calcd. for C_13_H_8_F_3_N_3_OSSe: C: 40.01, H: 2.07, N: 10.77, F: 13.63, S: 8.22, Se: 20.23; Found: C: 39.95, H: 2.06, N: 10.74, S: 8.25.

##### (E)-ethyl 3-amino-5-((4-hydroxy-2-methylphenyl) diazenyl)-4,6-dimethyl-2,3,7,7a-tetrahydroselenopheno[2,3-b] pyridine-2-carboxylate (16d)

Solid, brownish; yield: 90%; m.p. (°C): 740; IR (KBr) ν cm^−1^: 3,200, 3,400 (NH_2_); 1,645 (C=O;) 3,195 (N–H), 3,500 (O–H); ^1^H NMR (CDCl_3_): (δ ppm) (d, *J* = 15.9 Hz, 1H), 7.11–7.05 (m, 1H), 6.85–6.78 (m, 1H), 6.70–6.64 (m, 2H), 5.78 (s, 2H), 5.73 (s, 1H), 2.53 (s, 3H), 2.45 (q, *J* = 8.0 Hz, 2H), 2.34 (s, 3H), 1.14 (t, *J* = 8.0 Hz, 3H). ^13^C NMR (CDCl_3_): (δ ppm) δ 173.13, 159.00, 155.54, 143.31, 139.55, 137.11, 133.35, 131.48, 129.04, 128.72, 127.70, 125.96, 125.94, 123.43, 117.02, 113.60, 27.47, 23.03, 19.05, 16.44, 8.84; ESI MS (*m/z*): 436.35 [M+H]^+^. Anal. calcd. for C_19_H_24_N_4_O_3_Se: C: 52.41, H: 5.56, N: 12.87, Se: 18.14; Found: C: 52.36, H: 5.55, N: 12.84.

### Biological Evaluation

#### Antibacterial and Antifungal Activities

The selenopheno[2,3-b]pyridine derivatives were screened for their antimicrobial performance against Gram positive (*S2014-Staphylococcus aureus, S5265 Streptococcus pyogenes*), Gram negative (*Escherichia coli* K12, *Pseudomonas aeruginosa NACRES NA.24*) bacterial strains, and for their antifungal activity (*Candida albicans, VT000542, Aspergillus niger RMF02275L, Aspergillus clavatus, RMF02275L*). The investigation was carried out by the agar diffusion method with slight modification (Matar et al., 2003; Moustafa, 2005). The tested selenopheno[2,3-b]pyridine derivatives were added directly to the culture media, and the percentage growth inhibition was assessed after 3 days. The selenopheno[2,3-b]pyridine derivatives were prepared in DMSO in concentration of 400 μg/ml and sterilized by filtration through 0.22 μm sterilizing Millipore express filter. Negative controls were prepared using only DMSO. Ciprofloxacin, gentamicin and griseofulvin were used as reference standards to determine the sensitivity of Gram-positive, Gram-negative bacterial and fungal strains, respectively. The inoculated plates were incubated at 37°C for 3 days. The growth inhibition percentage was estimated using the following equation:

$$\begin{array}{l} \%\text{Inhibition}=\frac{\left(d1-d2\;\right)}{d1}x100 \end{array}$$

Where d1 is the diameter of the bacterial colony (mm) in the negative control plates after 3 days, and d2 is the diameter of the colony (mm) of the treated plates after the same period.

### Molecular Docking Study

The crystal structures of the proteins identified for *Escherichia Coli* (1kzn) were obtained from the protein data bank. Water molecules around the duplex were removed, and hydrogen atoms were added. The parameters and charges were allocated with MMFF94x force field. After alpha-site spheres were generated using the site finder module of MOE, our compound was docked in the active site, using the DOCK module of MOE. The Dock scoring in MOE software was calculated by London dG scoring function and was refined using two different methods. The planarity of the system was maintained, and the best poses were analyzed for the best score (Lafitte et al., 2002; Abdellattif et al., 2020; Almehmadi et al., 2020; Hosny et al., 2020; Hussein et al., 2020; Hussien and Abdelaziz, 2020).

### *In-silico* ADME Study

A computational study of selenium compounds was performed for prediction of ADME properties by QikProp3.2 tool available in Schrödinger 9.0 version (USA) and Molinspiration online property calculation toolkit to get an idea whether the compound has optimum pharmacokinetic properties to enter higher phases of the drug development process or not. Molinspiration strategy may be described as a complex balance of various molecular properties and structural features which conclude whether the appropriate molecule is related to the known drugs. These properties, mainly hydrophobicity, electronic distribution, hydrogen bonding characteristics, molecule size and flexibility, and of course presence of various pharmacophoric features affect the performance of molecule in a living organism, including bioavailability, transport properties, affinity to proteins, reactivity, toxicity, metabolic stability, and many others.

The diversity of potential drug targets (of which each needs a different combination of matching molecular characteristics) is so enormous, that it is possible to find a common denominator for all of them and to express molecule drug-likeness by a single “magic number.” Simple count criteria (like limits for molecular weight, log P, or the number of hydrogen bond donors or acceptors) have also relatively limited applicability and are useful only to discard obvious non-drugs.

Molinspiration strategy which leads to success is not only a universal drug-likeness score but also focuses on particular drug classes and the development of specific activity scores for each of these classes. The method uses sophisticated Bayesian statistics to compare structures of typical ligands active on the particular target with structures of inactive molecules and to identify substructure characteristics (which in turn determine physicochemical properties) representative for active molecules (Lipinski et al., 1997; Ertl et al., 2000; Veber et al., 2002).

## Conclusion

The designed seleno-pyridine derivatives were efficiently synthesized in satisfactory yields under the stated reaction conditions through the exploitation of the microwave assisted green synthesis and screened for their antibacterial and antifungal potential. The study results revealed that selected compounds exhibited good-to-significant activity. Among these, compound **14f** was found to be the most potent antibacterial agent as well as a good antifungal agent, which could serve as the lead compound. Furthermore, compound **14f** displayed the highest energy interaction within the binding pocket in the molecular docking study. In sight of these facts, compound **14f** could be the focus of further investigations for potential antimicrobial agents and sustenance for the design of new molecules. Finally, the DFT calculations were conducted to the molecular docking and the antimicrobial activity to give a complete investigation in terms of the chemical reactivity descriptors. The data revealed that the lower hydrophobicity, higher topological polar surface area, lower dipole moment, high H-bonding acceptor and donor, and *in*-*silico* absorption percentage of **14f** explicates its highest biological activity.

## Data Availability Statement

The original contributions presented in the study are included in the article/Supplementary Material, further inquiries can be directed to the corresponding authors.

## Author Contributions

MMHA suggested the idea and interpret spectroscopic data. MHA interpreted the results obtained from docking, performed the chemistry of the article, writing the draft, and validate the article. AAHA-R performed the arrangement of the manuscript and wrote the final form. AA performed English editing and validate the article. SM performed biological investigation. MAH performed chemistry and molecular docking studies. RO performed chemistry, edited English language, and discussed the results and commented on the manuscript. MH performed theoretical chemistry, calculations, and discussed the results. Finally, TA analyzed the data, edited English language, and discussed the results and commented on the manuscript. All authors contributed to the article and approved the submitted version.

## Conflict of Interest

The authors declare that the research was conducted in the absence of any commercial or financial relationships that could be construed as a potential conflict of interest.
