# Supplementary material for: Novel 2-Hydroselenonicotinonitriles and Selenopheno[2, 3-b]pyridines: Efficient Synthesis, Molecular Docking-DFT Modeling, and Antimicrobial Assessment
Source: Front Chem. 2021 May 10;9:672503. doi: 10.3389/fchem.2021.672503 (PMC8141565; doi:10.3389/fchem.2021.672503)
Supplement: Supplementary file 1 [file Data_Sheet_1.docx]

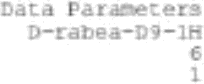


-0.09

-0.t2

-0.15


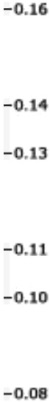


**Supplementary Data**

**Novel 2-Hydroselenonicotinonitriles and Selenopheno[2, 3-b]pyridines: Efficient Synthesis, Molecular Docking-DFT Modelling, and Antimicrobial Assessment**

Magda H. Abdellattif ^1*^, Adel A.H. Abdel-Rahman ^2^, Mohamed Mohamed Helmy Arief ^3^, Samar M Mounir ^4^, Amena Ali ^5^, Mostafa. A. Hussien ^6,7^, Rawda M. Okasha^8^, Tarek H. Afifi^8*^,

Mohamed Hagar ^9,10^

1. *Department of Chemistry, College of Science, Deanship of Scientific Research, Taif University, Al-Haweiah, P.O. Box 11099, Taif 21944, Saudi Arabia*
2. *Chemistry Department, Faculty of science, Menaufia University, Shebin Elkom, Egypt, Post no.00123,* [*adelnassar63@science-mnf.eg*](mailto:adelnassar63@science-mnf.eg)
3. *Chemistry Department, Faculty of Science, Benha University, Benha, Egypt,* [*mmharied@gmail.com*](mailto:mmharied@gmail.com)
4. *Department of Pharmacology,Faculty of Veterinary Medicine,Cairo University,12211,Egypt,* [*samar_mouneir@yahoo.com*](mailto:samar_mouneir@yahoo.com)
5. *Department of Pharmaceutical Chemistry, College of Pharmacy, Taif University, P.O.Box 11099, Taif, 21944 Saudi Arabia.* [*amrathore@tu.edu.sa*](mailto:amrathore@tu.edu.sa) *+966 592899232*
6. *Department of Chemistry, Faculty of Science, King Abdulaziz University, P.O. Box 80203 Jeddah 21589, Saudi Arabia*
7. *Department of Chemistry, Faculty of Science, Port Said University, Port Said, 42521, Egypt*
8. *Department of Chemistry, Faculty of Science, Taibah University, Al-Madinah Al-Munawarah 30002, Saudi Arabia,* [*rawdao@yahoo.com*](mailto:rawdao@yahoo.com) *,* [*afifith@yahoo.com*](mailto:afifith@yahoo.com)
9. *Chemistry Department, College of Sciences, Yanbu, Taibah University, Yanbu, 30799, Saudi Arabia, mhagar@taibahu.edu.sa.*
10. *Chemistry Department, Faculty of Science, Alexandria University, Alexandria 21321, Egypt.*

*****Corresponding authors: Email address: [m.hasan@tu.edu.sa](mailto:m.hasan@tu.edu.sa); [afifith@yahoo.com](mailto:afifith@yahoo.com)

**Experimental**

**General**

Melting point determination was done using open capillary tubes on an electrical melting point apparatus. Bruker spectrospin DPX-400MHz was used to record the ^1^H NMR and ^13^C NMR spectra. Chemical shift (δ) values were stated in parts per million (ppm) using internal standard tetramethylsilane. The D_2_O exchange confirmed the exchangeable protons (OH and NH). LC–MS/MS (PerkinElmer) was used to record the mass spectra, presented as *m/z*. Elemental analyses were achieved by using PerkinElmer 240 analyzer. The purity of synthesized compounds as well as progress of reaction were assessed by ascending thin layer chromatography (TLC) (silica gel G) by using methanol/chloroform (9:1 v/v) and methylene chloride/chloroform (4:1 v/v) combination as solvent system.

**Biological Evaluation**

***Antibacterial and antifungal activities***

The Selenopheno[2,3-b]pyridine derivatives were screened for their antimicrobial performance against Gram positive (*S. aureus*, *S. pyogenes*), Gram negative (*E. coli*, *P. aeruginosa*) bacterial strains, and for their antifungal activity (*C. albicans*, *A. nigar*, *A. clavatus*). The investigation was carried out by the agar diffusion method with slight modification ([Matar et al., 2003](#_ENREF_9)) ([Moustafa, 2005](#_ENREF_10)). The tested selenopheno[2,3-b]pyridine derivatives were added directly to the culture media, and the percentage growth inhibition was assessed after three days. The Selenopheno[2,3-b]pyridine derivatives were prepared in DMSO in concentration of 400 ug/ml and sterilized by filtration through 0.22 μm sterilizing Millipore express filter. Negative controls were prepared using only DMSO. Ciprofloxacin, gentamicin and Griseofulvin were used as reference standards to determine the sensitivity of Gram‑positive, Gram‑negative bacterial and fungal strains, respectively. The inoculated plates were incubated at 37°C for three days. The growth inhibition percentage was estimated using the following equation:

% Inhibition = $\frac{\left( d1-d2 \right)}{d1}x100$

where d1 is the diameter of the bacterial colony (mm) in the negative control plates after three days, and d2 is the diameter of the colony (mm) of the treated plates after the same period.

**Molecular docking study**

The crystal structures of the proteins identified for *Escherichia Coli* (1kzn) were obtained from the protein data bank. Water molecules around the duplex were removed, and hydrogen atoms were added. The parameters and charges were allocated with MMFF94x force field. After alpha-site spheres were generated using the site finder module of MOE, our compound was docked in the active site, using the DOCK module of MOE. The Dock scoring in MOE software was calculated by London dG scoring function and was refined using two different methods. The planarity of the system was maintained, and the best poses were analyzed for the best score ([Lafitte et al., 2002](#_ENREF_8)) ([Abdellattif et al., 2020](#_ENREF_1)) ([Almehmadi et al., 2020](#_ENREF_2)) ([Hosny et al., 2020](#_ENREF_5)) ([Hussein et al., 2020](#_ENREF_6)) ([Hussien and Abdelaziz, 2020](#_ENREF_7)).

**Computational methods and calculations**

The theoretical calculations for the investigated compounds were carried out by Gaussian 09 software ([Frisch et al., 2009](#_ENREF_4)). DFT/B3LYP methods using 6-31G (d, p) basis set was selected for the calculations. The geometries were optimized by minimizing the energies with respect to all geometrical parameters without imposing any molecular symmetry constraints. The structures of the optimized geometries had been drawn with Gauss Viewn. ([Dennington et al., 2009](#_ENREF_3)). Moreover, the calculated frequencies were carried out using the same level of theory. The frequency calculations showed that all structures were stationary points in the geometry optimization method with none imaginary frequency.


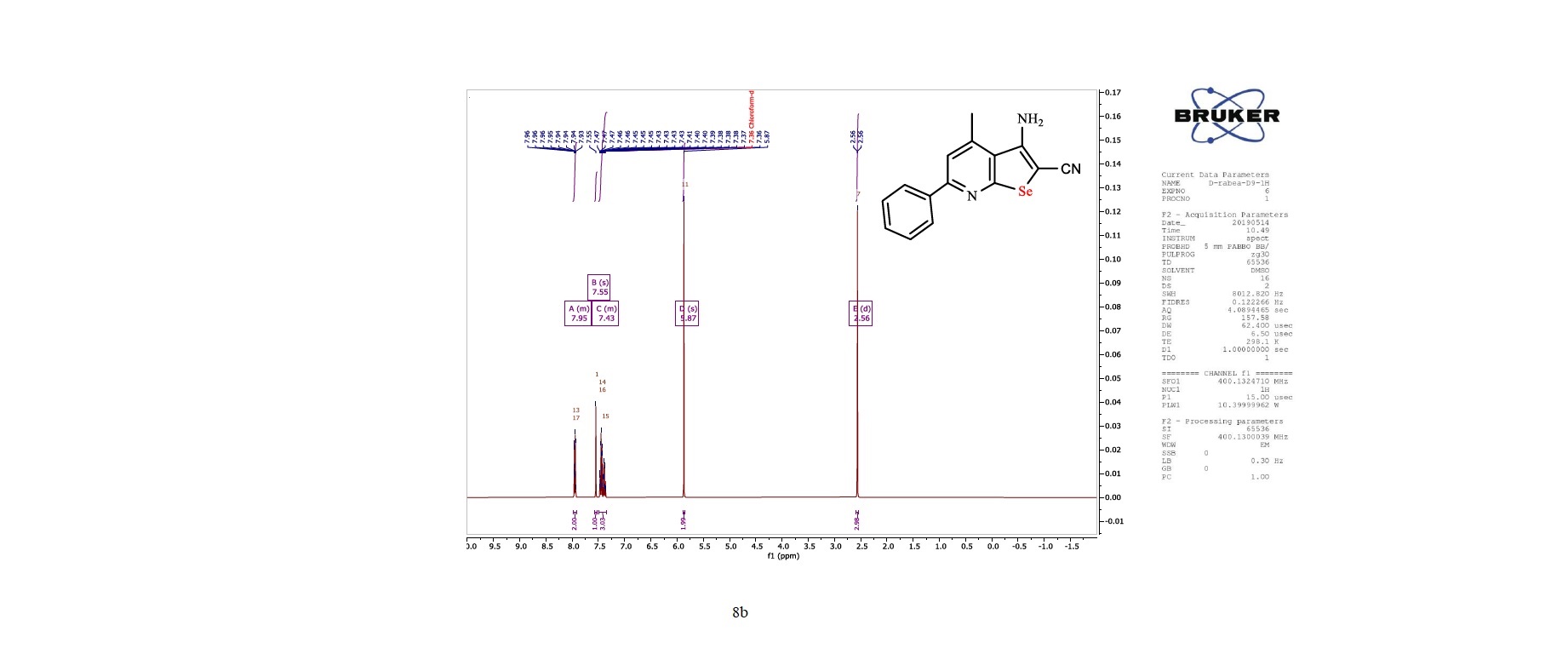


**Figure S1**. ^1^HNMR of Compound **8b**


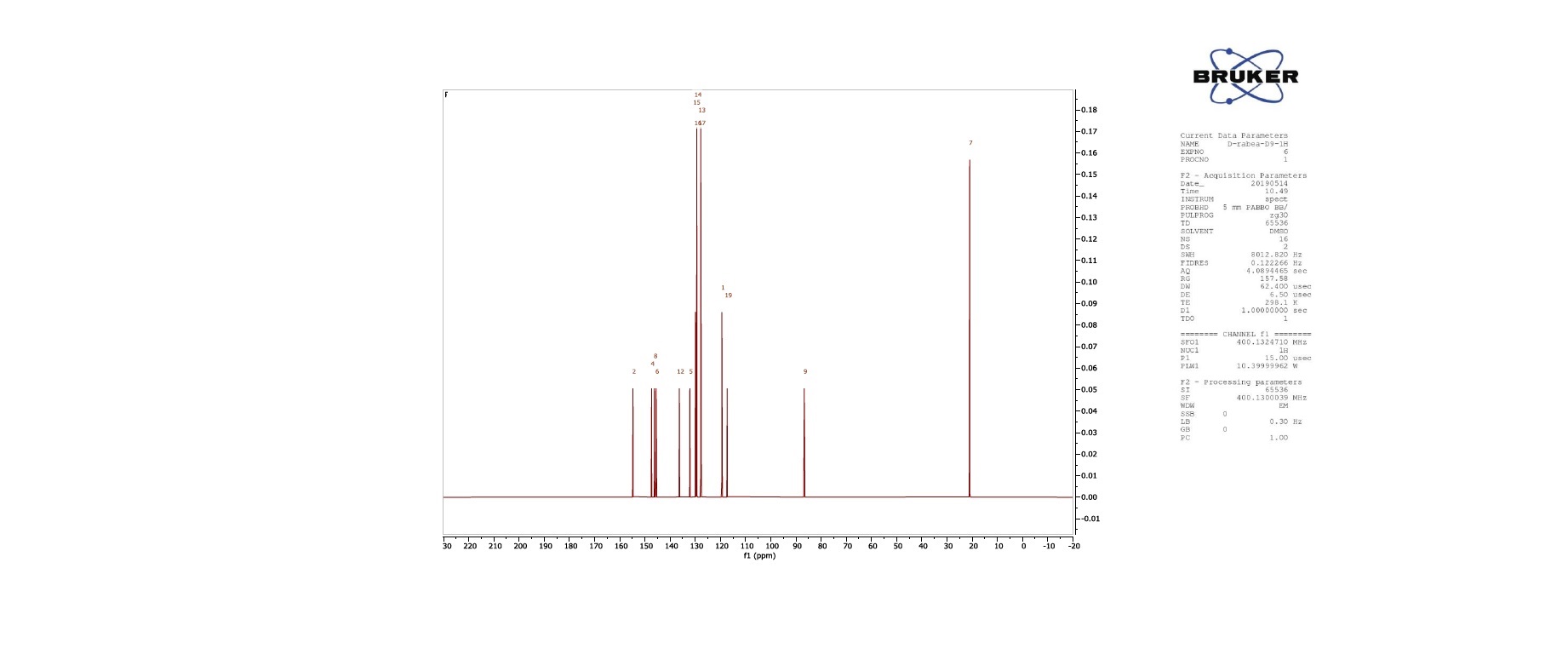


**Figure S2**. ^13^CNMR of Compound **8b**

**
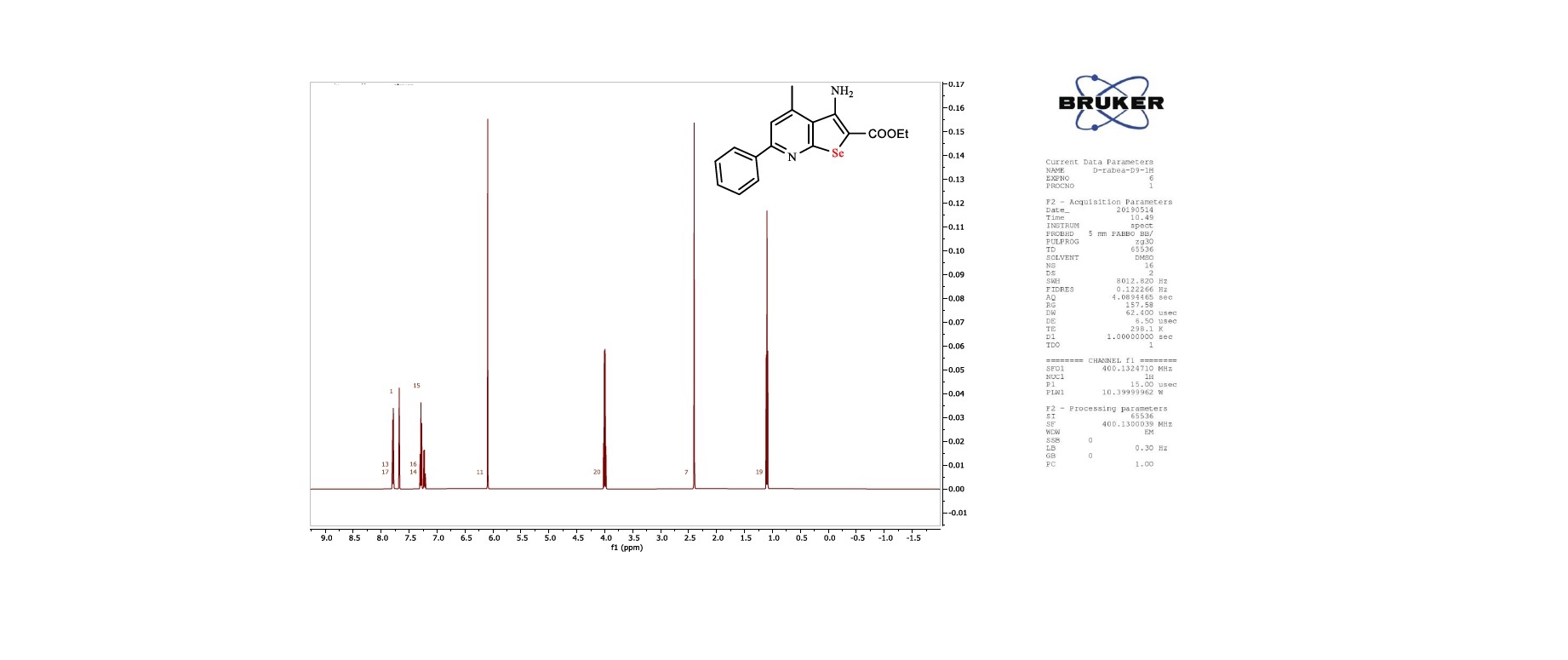
**

**Figure S3**. ^1^HNMR of Compound **9b**


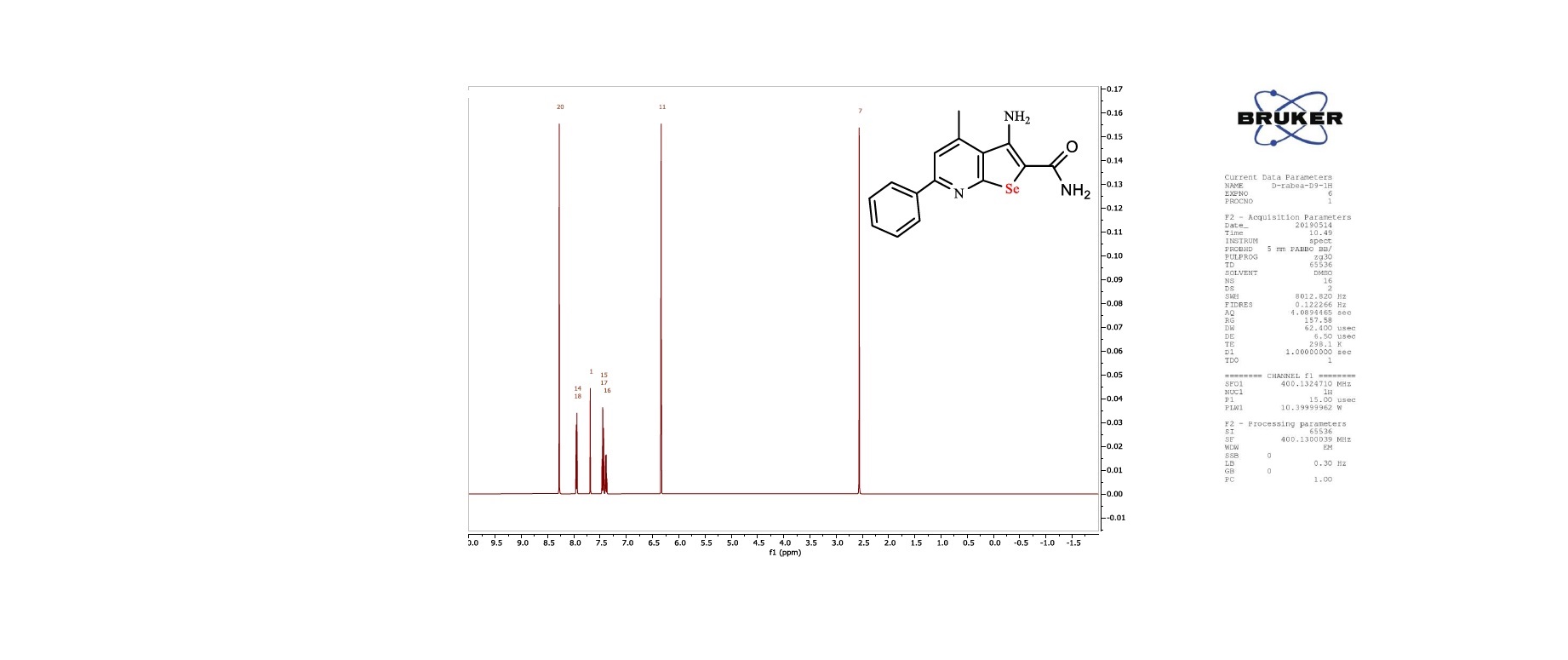


**Figure S3**. ^1^HNMR of Compound **10b**


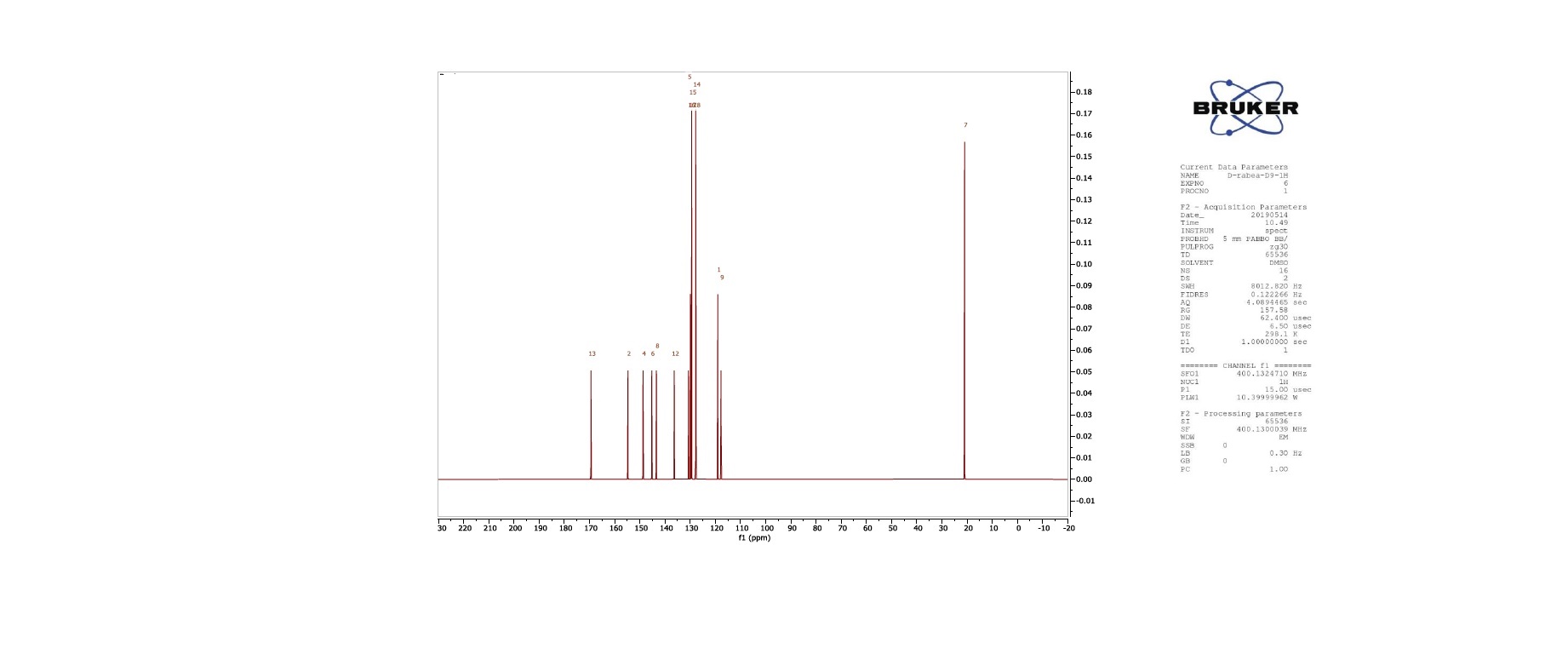


**Figure S4**. ^13^CNMR of Compound **10b**

**
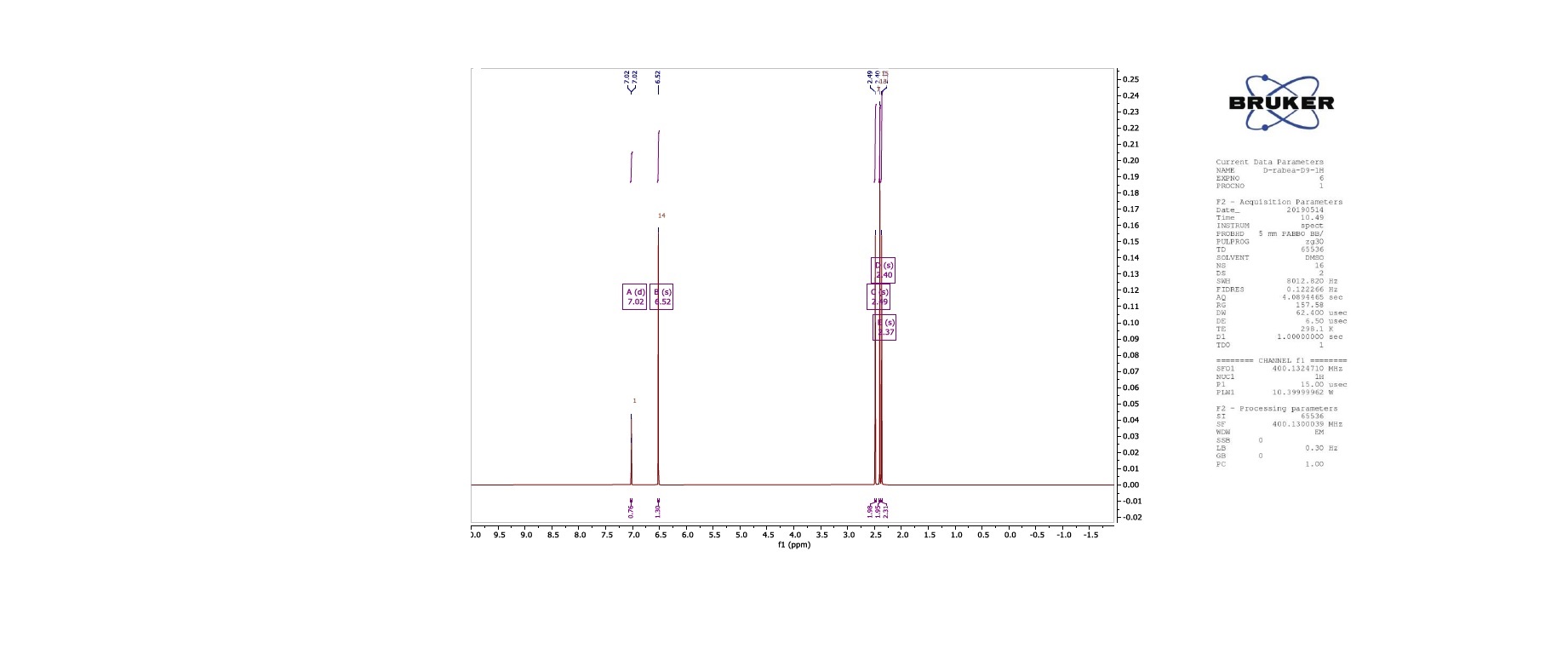
**

**Figure S5**. ^1^HNMR of Compound **12a**

**
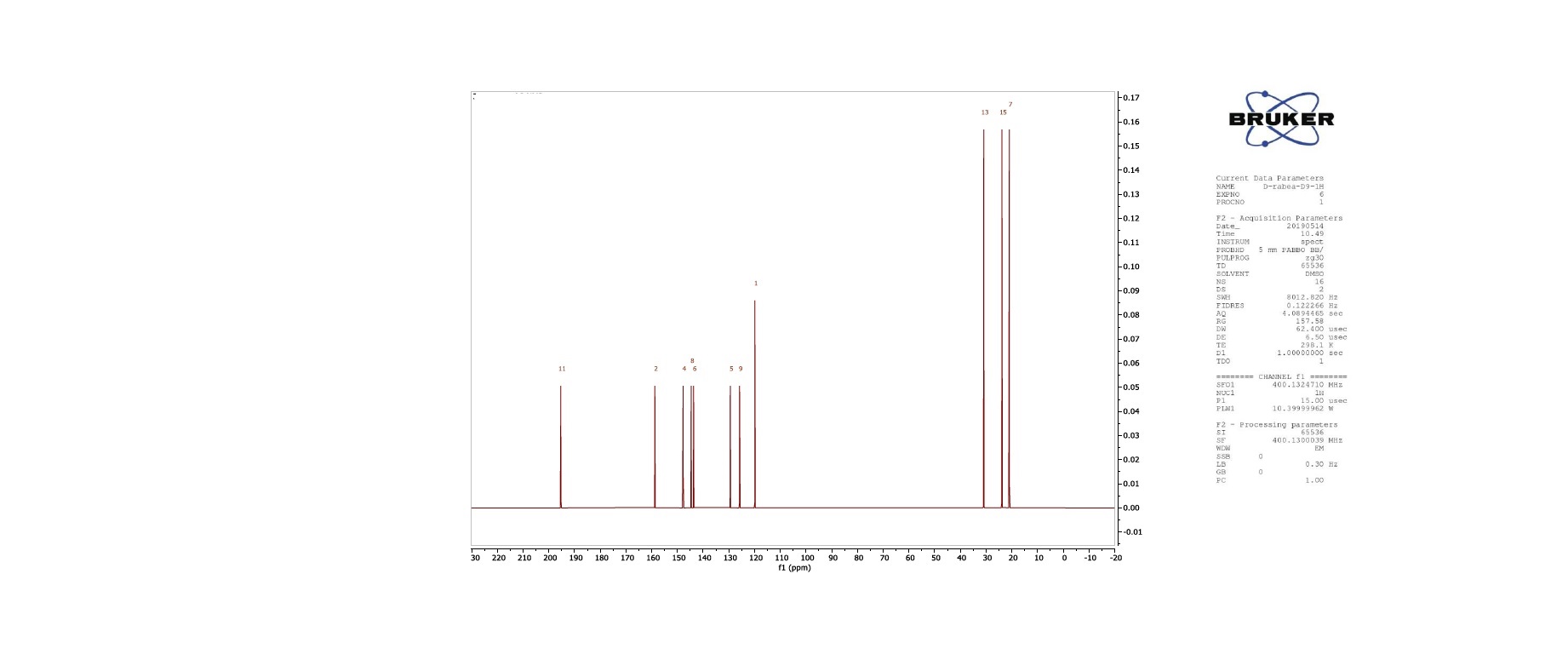
**

**Figure S6**. ^13^CNMR of Compound **12a**

**
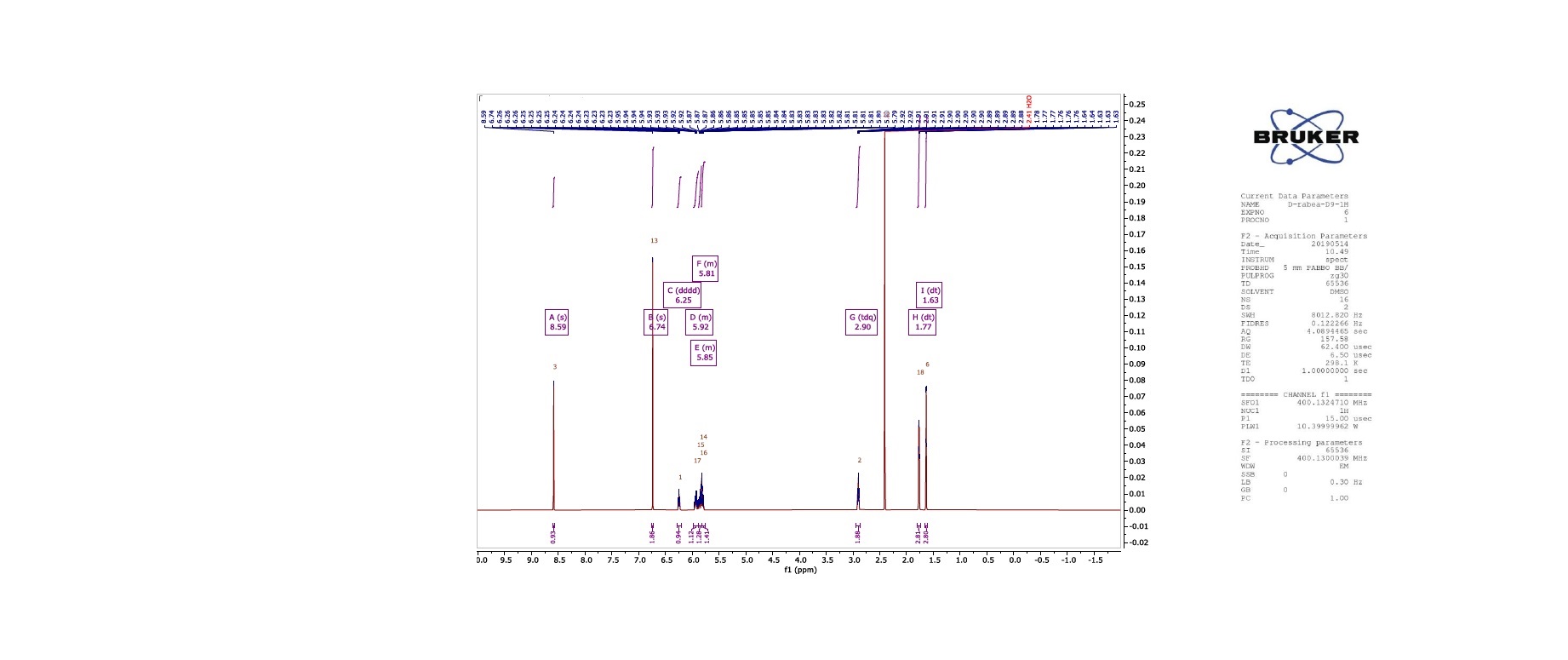
**

**Figure S7**. ^1^HNMR of Compound **12b**

**
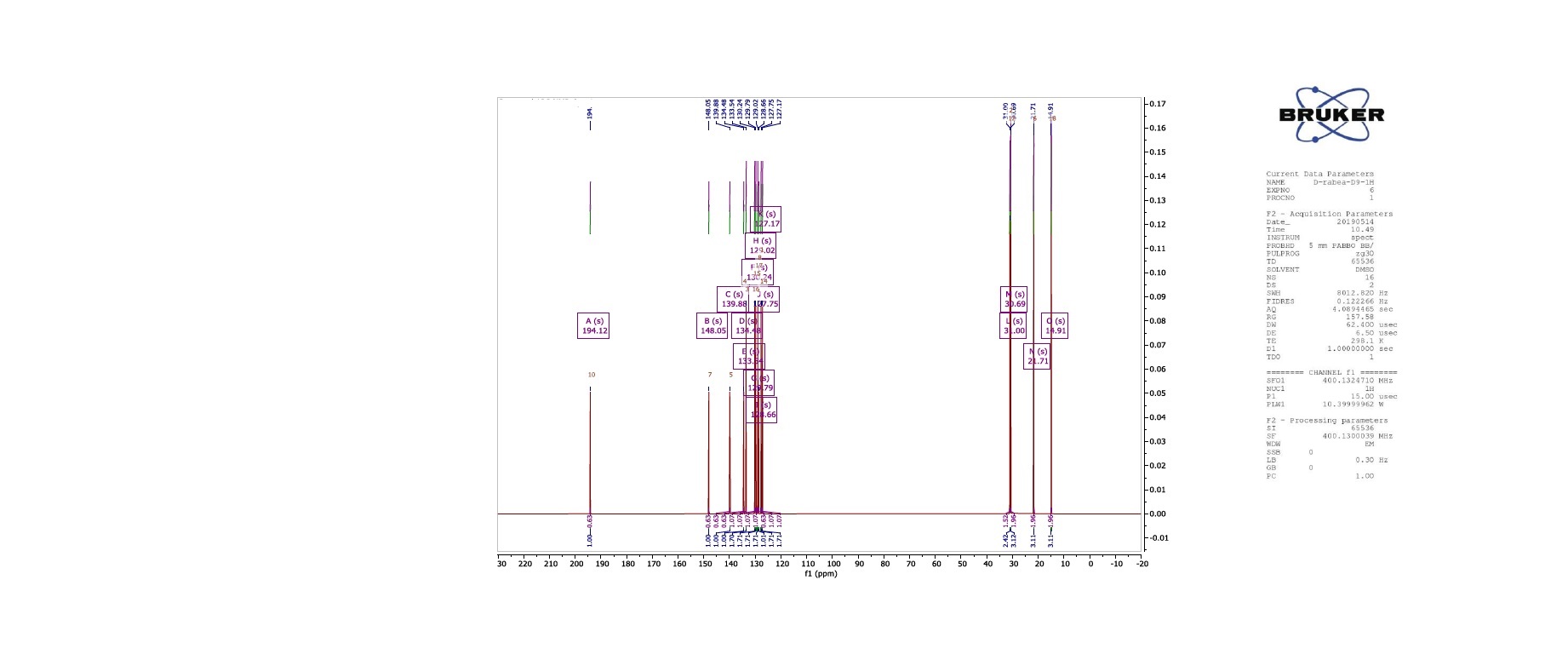
**

**Figure S8**. ^13^CNMR of Compound **12b**

**
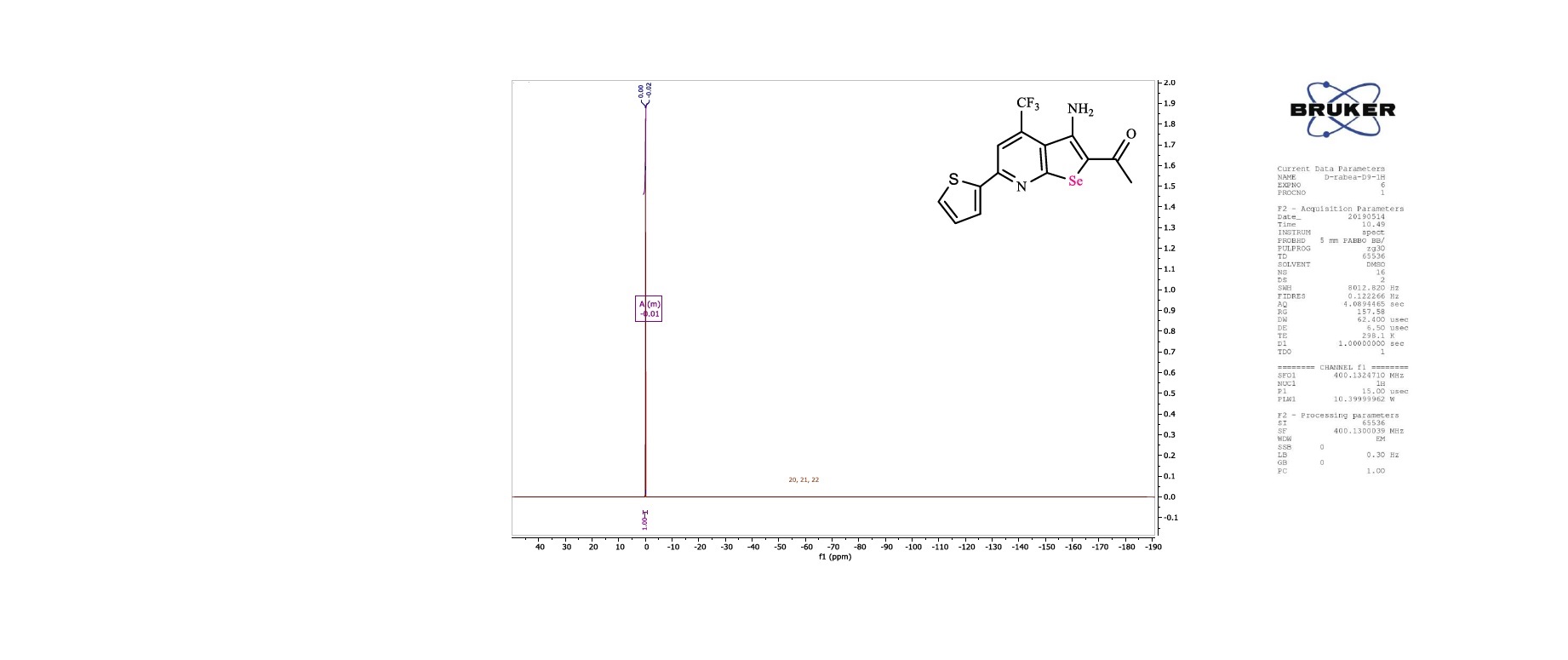
**

**Figure S9**. ^1^HNMR of Compound **12c**

**
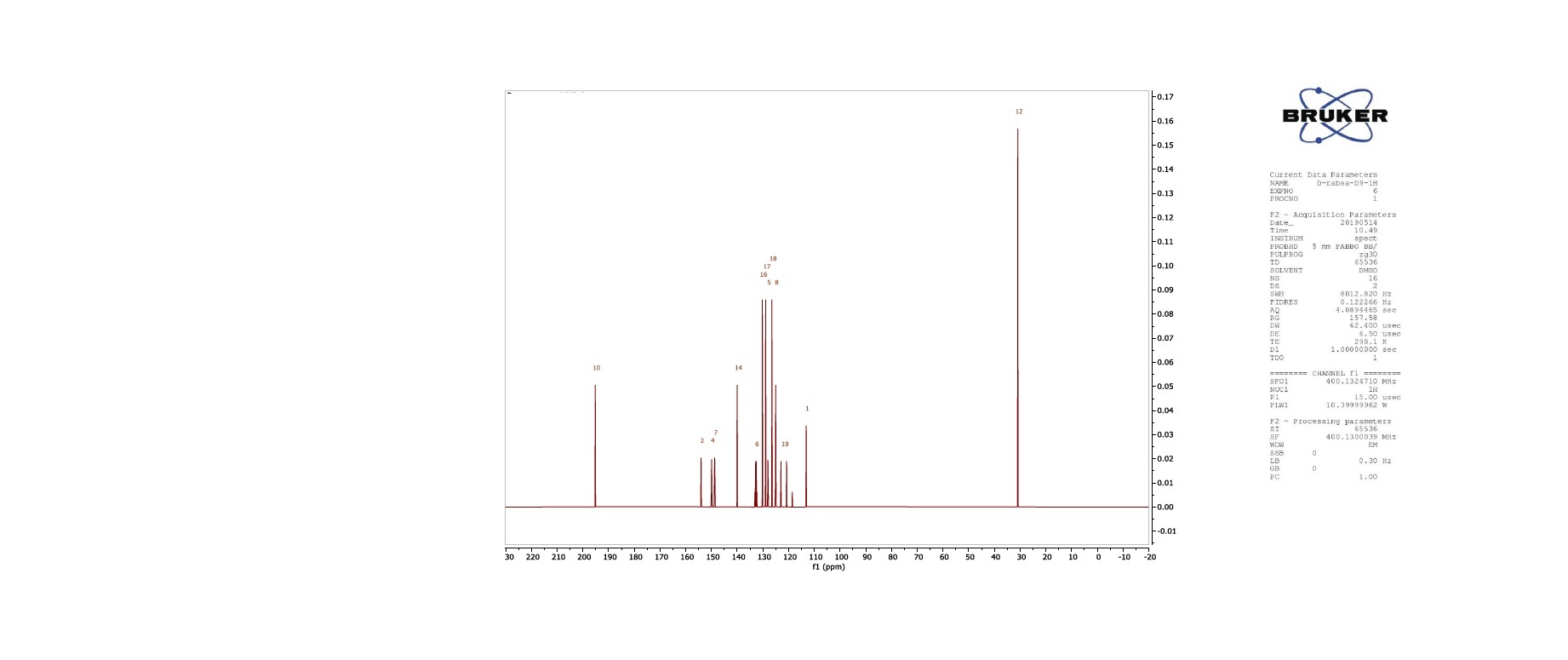
**

**Figure S10**. ^13^CNMR of Compound **12c**

**
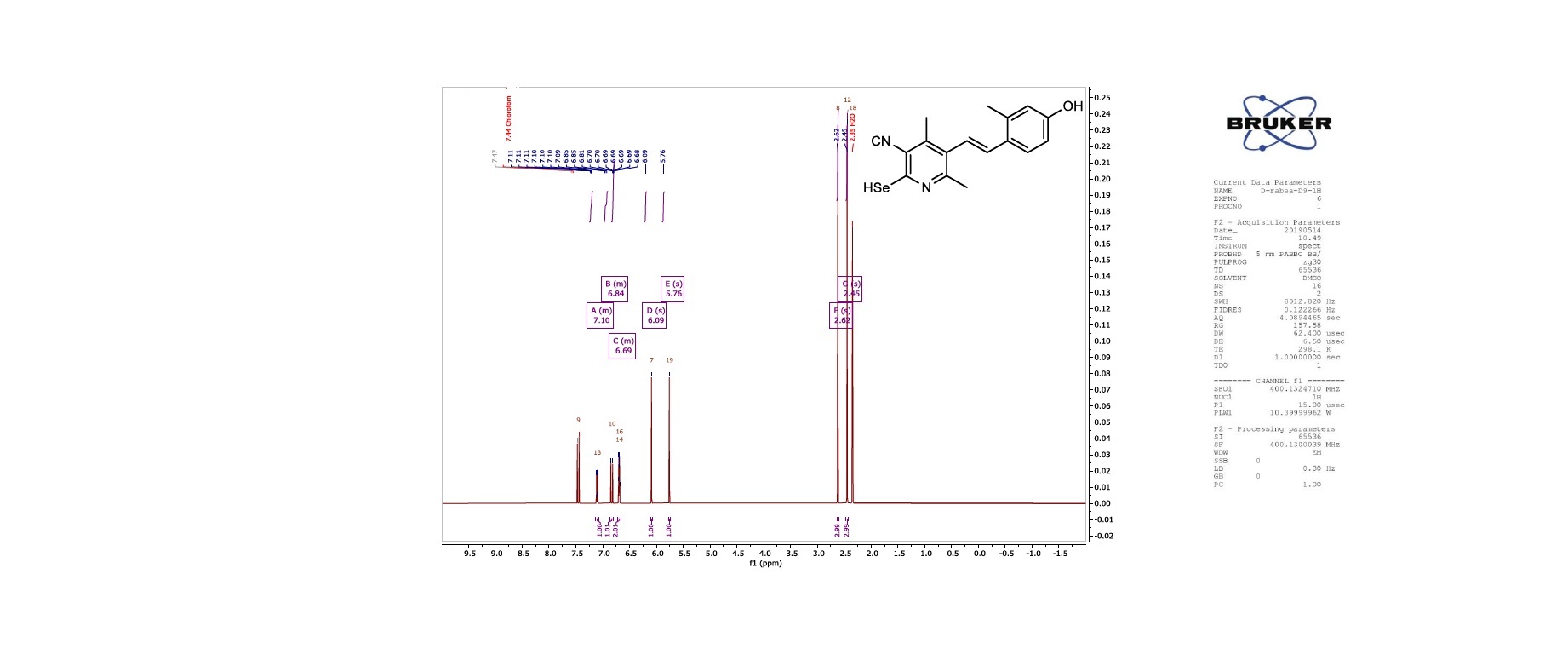
**

**Figure S11**. ^1^HNMR of Compound **14d**

**
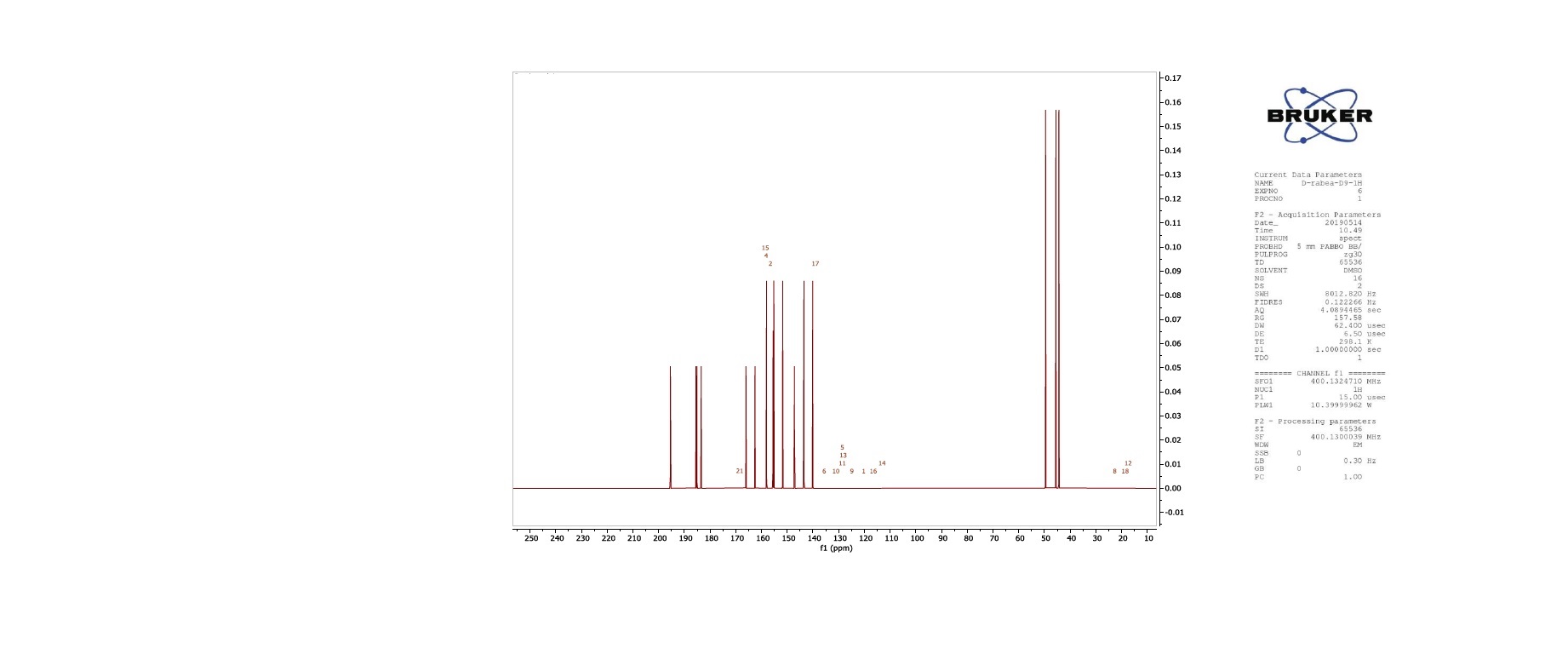
**

**Figure S12**. ^13^CNMR of Compound **14d**

**
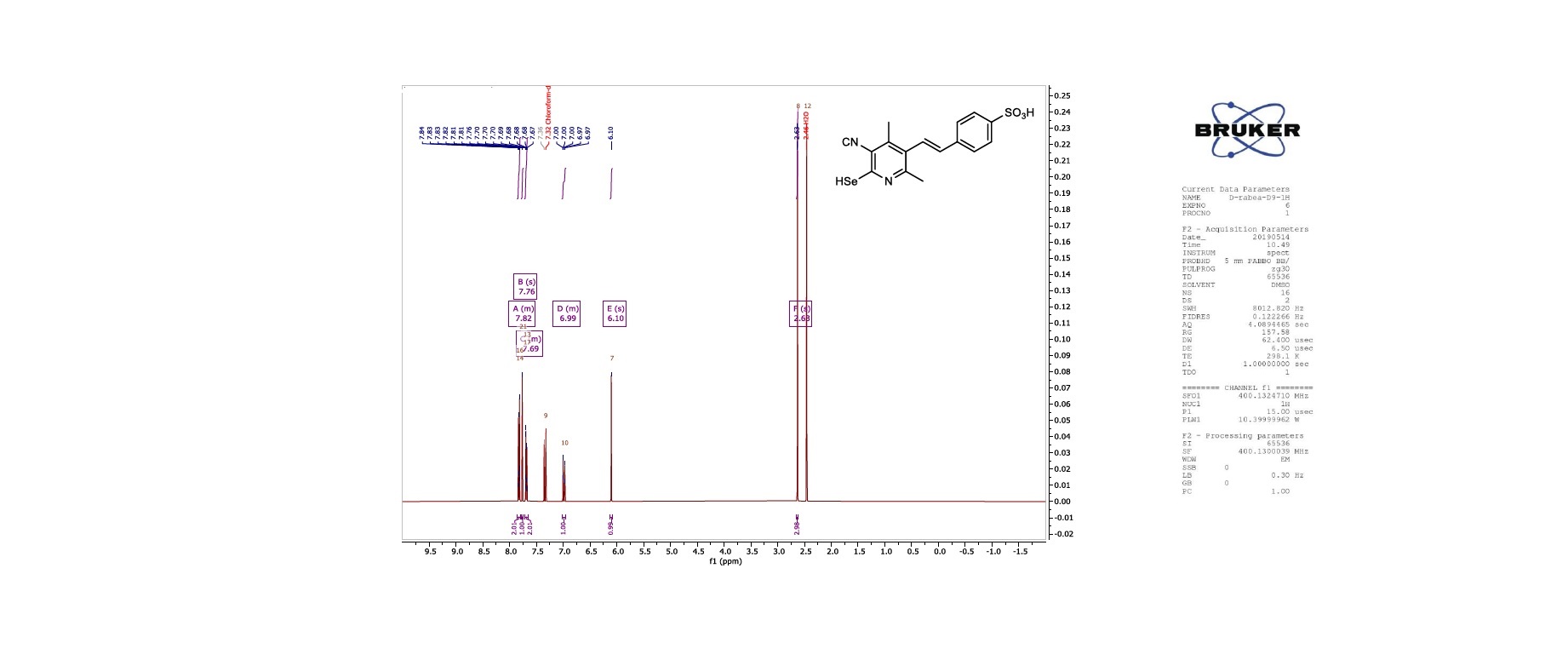
**

**Figure S13**. ^1^HNMR of Compound **14e**

**
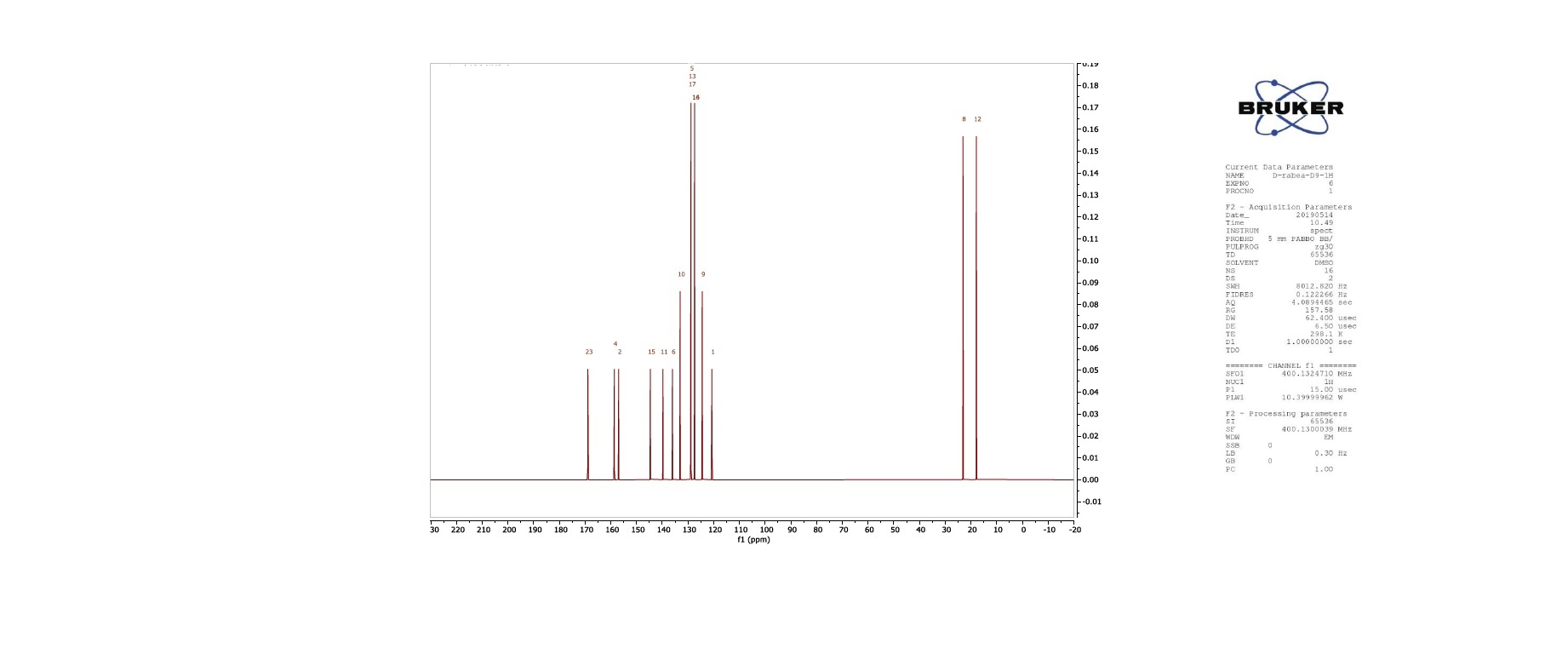
**

**Figure S14**. ^13^CNMR of Compound **14e**

**
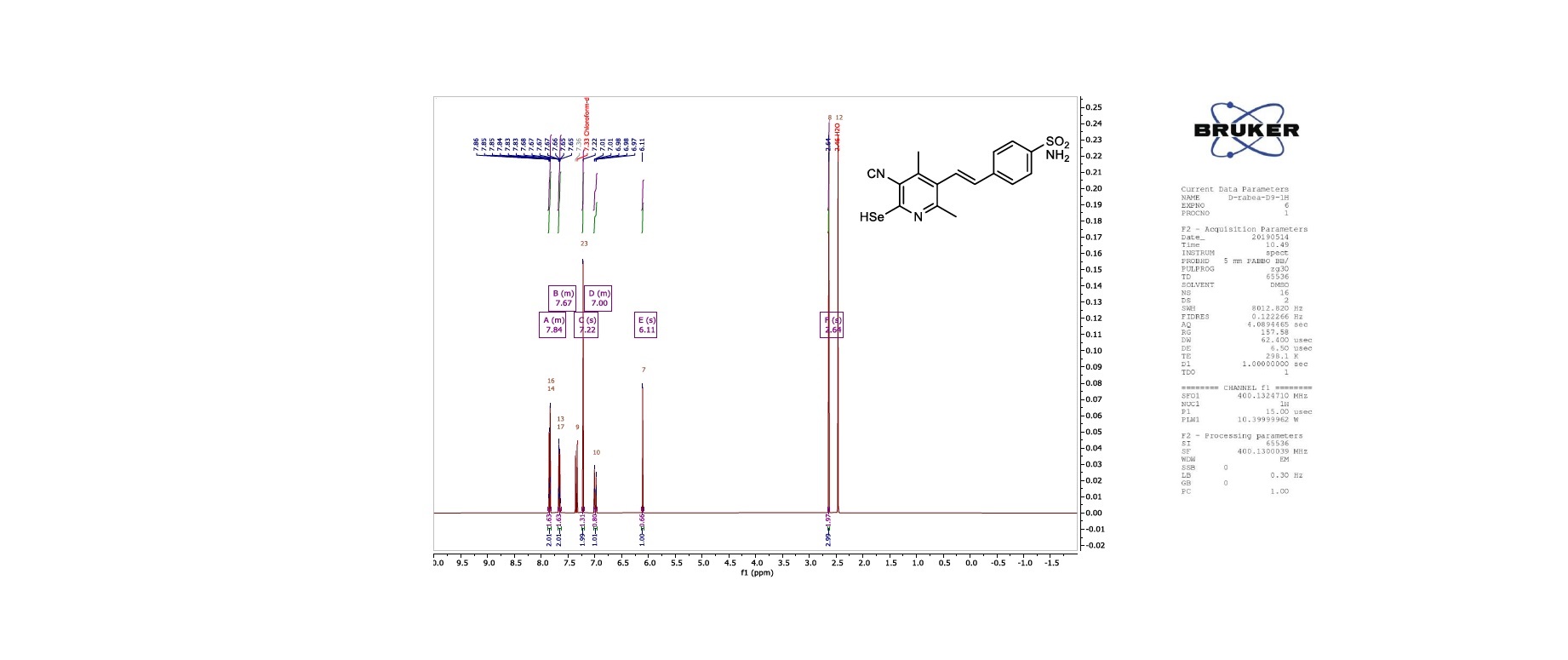
**

**Figure S15**. ^1^HNMR of Compound **14f**

**
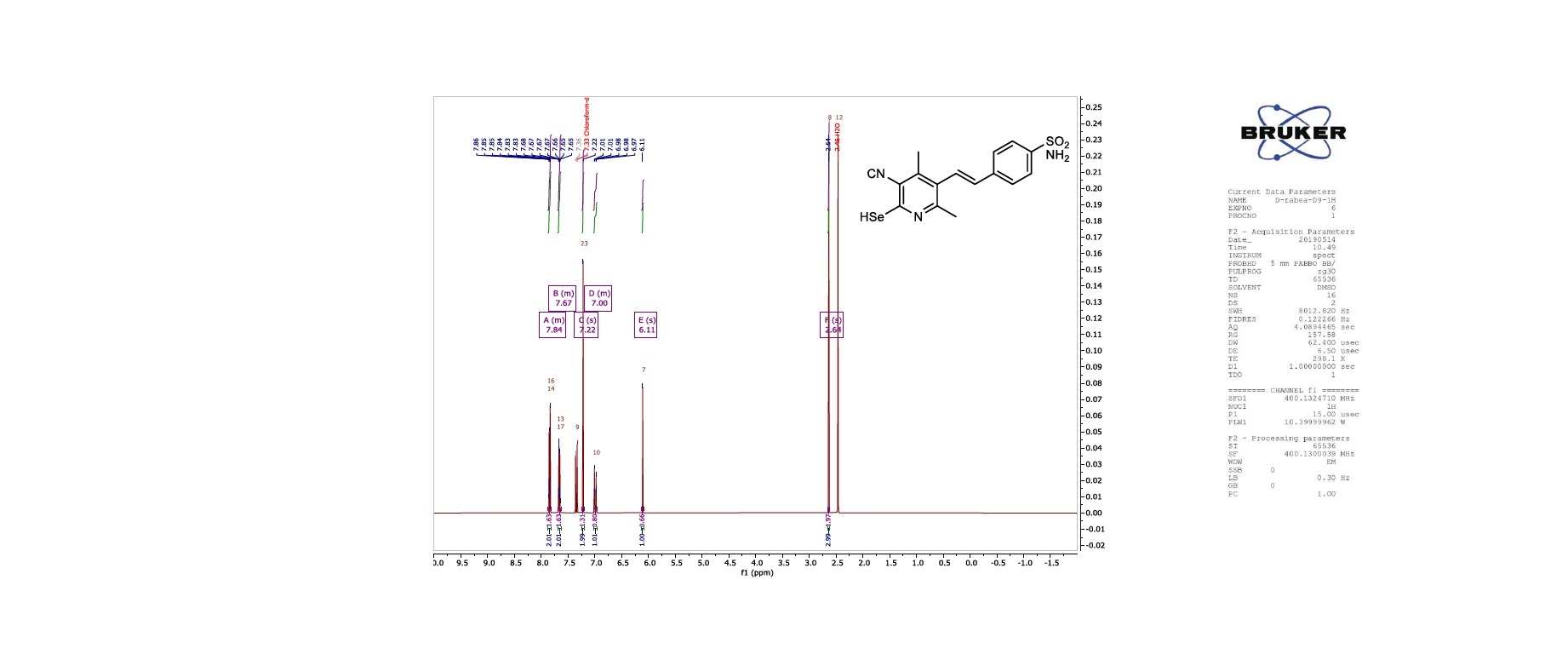
**

**Figure S16**. ^1^HNMR of Compound **16d**

**
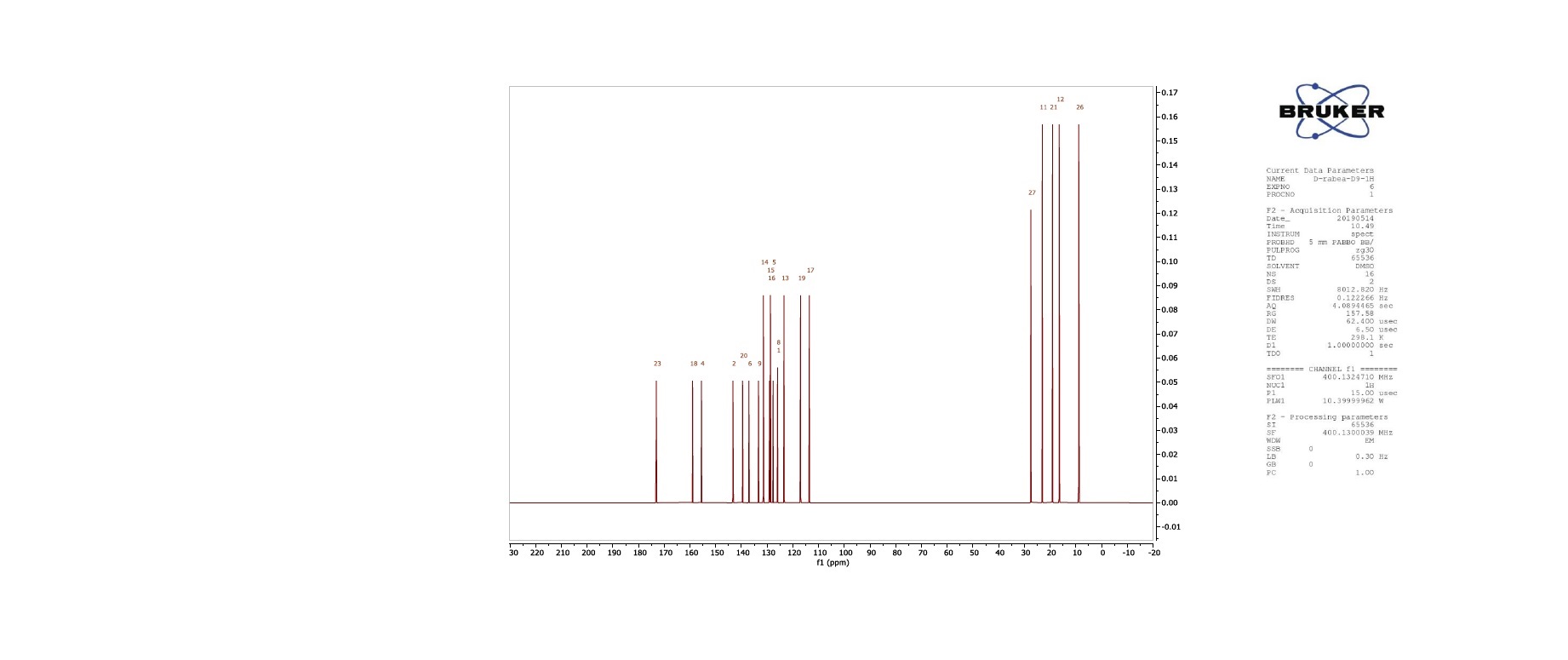
**

**Figure S17**. ^13^CNMR of Compound **16d**

**References**

Abdellattif, M.H., Ali, O.A., Arief, M.M., and Hussien, M.A. 2020. A One-pot Synthesis of Novel Derivatives of Oxadiazine-4-thione, and its Antibacterial Activity, and Molecular Modeling Studies. *Current organic synthesis* 17 pp 230-242.

Almehmadi, S.J., Alamry, K.A., Elfaky, M., Asiri, A.M., Hussien, M.A., Al-Sheheri, S., and Hussein, M.A. 2020. The role of the arylidene linkage on the antimicrobial enhancement of new tert-butylcyclohexanone-based polyketones. *Polymer Bulletin* pp 1-21.

Dennington, R., Keith, T., and Millam, J. 2009. GaussView, version 5.

Frisch, M., Trucks, G., Schlegel, H., Scuseria, G., Robb, M., Cheeseman, J., Scalmani, G., Barone, V., Mennucci, B., and Petersson, G. 2009. Gaussian Inc. *Wallingford Ct* 2009.

Hosny, N.M., Hussien, M.A., Motawa, R., Belal, A., and Abdel‐Rhman, M.H. 2020. Synthesis, Spectral, Modeling, Docking and Cytotoxicity Studies on 2‐(2‐aminobenzoyl)‐N‐ethylhydrazine‐1‐carbothioamide and its divalent metal complexes. *Applied Organometallic Chemistry* 34 pp e5922.

Hussein, M.A., Alamry, K.A., Almehmadi, S.J., Elfaky, M., Džudžević-Čančar, H., Asiri, A.M., and Hussien, M.A. 2020. Novel biologically active polyurea derivatives and its TiO2-doped nanocomposites. *Designed monomers and polymers* 23 pp 59-74.

Hussien, M.A., and Abdelaziz, A.E. 2020. Molecular docking suggests repurposing of brincidofovir as a potential drug targeting SARS-CoV-2 ACE2 receptor and main protease. *Network Modeling Analysis in Health Informatics and Bioinformatics* 9 pp 1-18.

Lafitte, D., Lamour, V., Tsvetkov, P.O., Makarov, A.A., Klich, M., Deprez, P., Moras, D., Briand, C., and Gilli, R. 2002. DNA gyrase interaction with coumarin-based inhibitors: the role of the hydroxybenzoate isopentenyl moiety and the 5 ‘-methyl group of the noviose. *Biochemistry* 41 pp 7217-7223.

Matar, M.J., Ostrosky-Zeichner, L., Paetznick, V.L., Rodriguez, J.R., Chen, E., and Rex, J.H. 2003. Correlation between E-test, disk diffusion, and microdilution methods for antifungal susceptibility testing of fluconazole and voriconazole. *Antimicrobial agents and chemotherapy* 47 pp 1647-1651.

Moustafa, M. 2005. Synthesis and Structural and Biological Activity Studies on Some Lanthanide Chelates with O‐and N‐Containing Ligands. *Spectroscopy letters* 38 pp 23-34.
